# Adoptive transfer of VSIG4^+^ macrophages interrupts the CCL2-CCR2 inflammatory amplification loop to mitigate acetaminophen-induced acute liver injury in murine and human organoid models

**DOI:** 10.1186/s12967-026-08631-y

**Published:** 2026-07-20

**Authors:** Biao Duan, Jun Chen, Guan Liu, Jiacheng Lin, Shiyu Yang, Xiaoni Kong, Weifang Rong, Weifeng Tan

**Affiliations:** 1 Hepatobiliary Surgery Center, Tongji Hospital, School of Medicine, Tongji University, Shanghai, 200092 China; 2 Baoshan Branch of Renji Hospital, Shanghai Jiao Tong University School of Medicine, Shanghai, 200444 China; 3 Shenzhen Institutes of Advanced Technology, Chinese Academy of Sciences, Shenzhen, 518055 China; 4 Central Laboratory, Shuguang Hospital Affiliated to Shanghai University of Traditional Chinese Medicine, Shanghai, 201203 China; 5 Department of Gastroenterology, Songjiang Hospital Affiliated to Shanghai Jiao Tong University School of Medicine, Shanghai, 201600 China

**Keywords:** Acute liver injury, VSIG4^+^ macrophages, Adoptive cell therapy, CCL2-CCR2 axis, Chemotaxis, Human liver organoids

## Abstract

**Background:**

Acute liver injury (ALI) can rapidly progress to life-threatening acute liver failure. Liver transplantation remains the only definitive treatment, despite critical donor shortages. Macrophage-based therapies have shown promise in ALI but face challenges related to subset imprecision and limited humanized validation. VSIG4 is generally considered a marker of Kupffer cells; however, it is also expressed in monocyte-derived macrophages (MoMFs), and its role remains unclear during ALI.

**Methods:**

By integrating single-cell RNA sequencing (scRNA-seq) data from human and mouse liver tissues with clinical ALI tissue samples, we elucidated the dynamic changes in VSIG4^+^ macrophages (VSIG4^+^ Mφ) within the liver. We developed a reversible immunomagnetic nanoparticle system for the non-destructive isolation of viable VSIG4^+^ Mφ. The therapeutic efficacy and potential mechanisms of VSIG4^+^ Mφ were evaluated through tissue and molecular-level analyses in an acetaminophen (APAP)-induced ALI mouse model, as well as in a newly established vascularized human liver organoid ALI and monocyte chemotaxis model.

**Results:**

scRNA-seq revealed a previously underrecognized subpopulation of VSIG4^+^ MoMFs, which increases following ALI, while the number of resident VSIG4^+^ Kupffer cells decreases significantly. Clinical ALI tissue samples also confirmed the presence of CCR2^+^VSIG4^+^ cells in the livers of patients with ALI. In the APAP-induced ALI animal model, adoptive transfer of isolated VSIG4^+^ MoMFs dramatically decreased serum alanine transaminase, hepatic necrosis, and apoptosis while increasing anti-inflammatory cytokines. In contrast, adoptive transfer of unselected BMDM failed to improve liver injury and instead exacerbated certain pro-inflammatory responses, including elevated TNF-α and reduced CD206 expression. Mechanistically, VSIG4^+^ MoMFs prevented inflammatory amplification by suppressing NF-κB-dependent CCL2 transcription, thereby disrupting the CCL2-CCR2 chemotactic axis and reducing pro-inflammatory CCR2^+^ monocyte and macrophage recruitment. We further developed vascularized human liver organoids and APAP-induced hepatocyte injury and monocyte chemotaxis, finding that the chemotaxis-interrupting mechanism was fully recapitulated in the human liver organoid ALI model.

**Discussion:**

This study identifies VSIG4^+^ MoMFs as a therapeutically viable subset for ALI, with clear superiority over unselected BMDM. By blocking the CCL2-CCR2 inflammatory amplification loop, these cells attenuate liver injury in both mouse and humanized models. These consistent findings provide robust preclinical evidence to support the advancement of VSIG4^+^ Mφ-based immunotherapies into clinical practice.

**Graphical Abstract:**

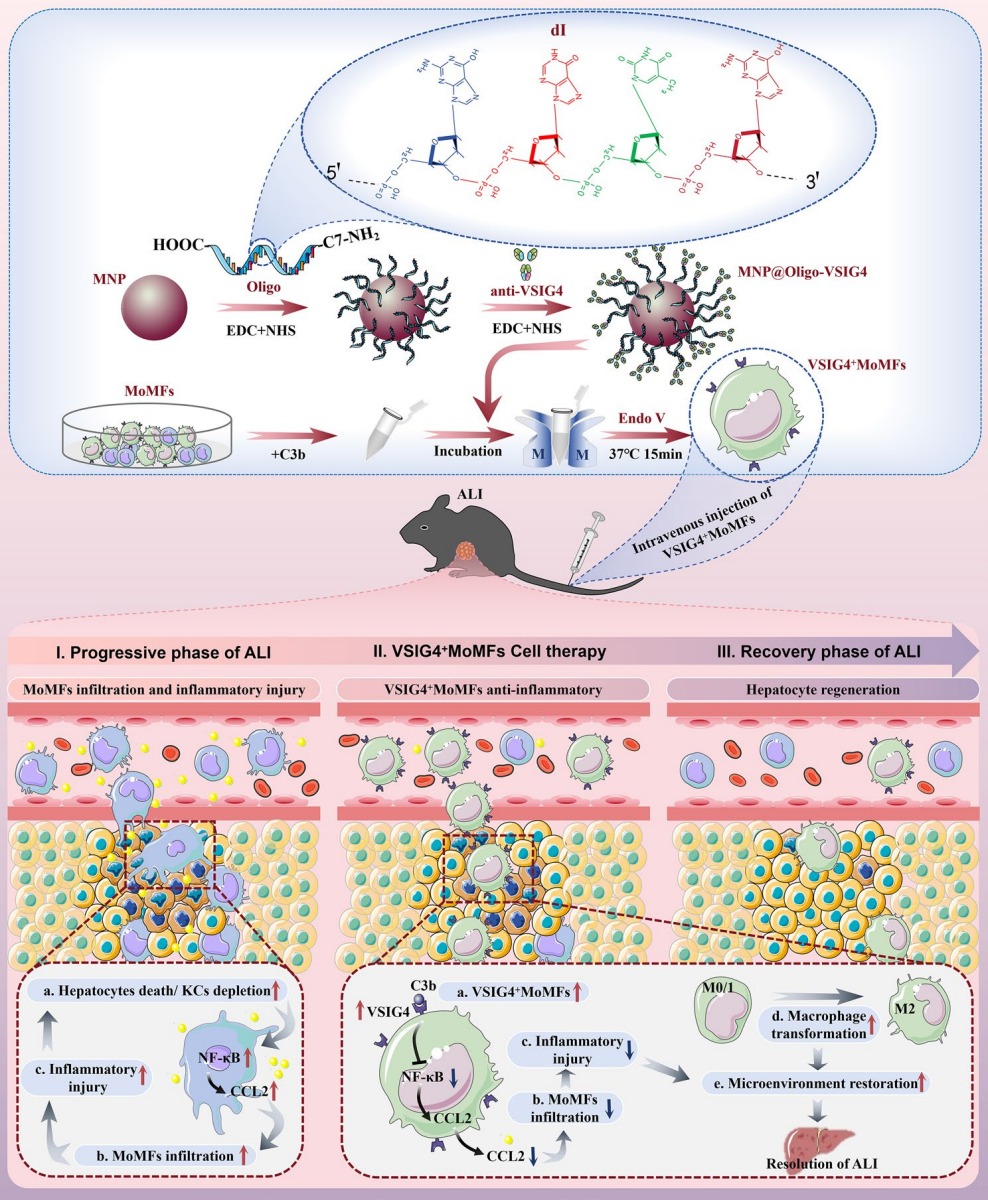

**Supplementary Information:**

The online version contains supplementary material available at 10.1186/s12967-026-08631-y.

## Background

Acute liver injury (ALI) represents a critical juncture in the spectrum of liver disease, with clinical outcomes polarized between resolution through the liver’s intrinsic regenerative capacity and rapid progression to life-threatening acute liver failure (ALF) [1–3]. This trajectory is often rapid: without liver transplantation, survival rates for patients with ALF range from only 27% to 40% [4]. Although emergency liver transplantation improves 1-year survival to approximately 80% [5], its application is severely constrained by a global shortage of donor organs, resulting in the death of nearly 16% of wait-listed patients [4]. These realities underscore the urgent need for effective therapies that can intercept the progression from ALI to ALF.

For end-stage liver diseases, cell therapy has become a promising treatment strategy. Hepatocyte-derived liver progenitor-like cells (HepLPCs) have been successfully transplanted for cirrhosis, and GMP-compliant, component-defined culture systems for biliary epithelial cells have been established, offering safe and scalable cell sources [6]. Strategies to repair the liver and avoid transplantation have gained significant attention in this context, especially those that target the hepatic immune microenvironment [7]. Macrophage polarization and reprogramming also have been therapeutically exploited in other inflammatory diseases, such as osteoarthritis, where engineered systems aim to shift the balance from pro-inflammatory to anti-inflammatory states [8].

Macrophages play dichotomous roles during liver injury: they can exacerbate inflammation in the early phase, whereas alternatively activated macrophages (AAMs) with reparative functions orchestrate resolution by phagocytosing cellular debris, secreting anti-inflammatory mediators, and promoting tissue regeneration [7]. A recent preclinical study demonstrated that, even when administered after the therapeutic window of N-acetylcysteine (NAC), cryopreserved human AAMs effectively reduced necrosis, modulated inflammation, and enhanced regeneration in acetaminophen (APAP)-induced ALI [9], confirming the feasibility of “off-the-shelf” macrophage-based therapies. Recent reviews have highlighted AAM-based therapy as an emerging and necessary option in ALI, with APAP-induced injury representing the most probable initial clinical application [10]. However, current AAM-based approaches face two fundamental challenges. First, the in vitro use of cytokine-induced AAMs (M2a) provides only an approximate indicator of cellular function. Differences between in vivo and in vitro environments can lead to phenotypic variations, complicating precise definition and reproducible production. Second, preclinical evaluation of AAM-based therapies relies primarily on mouse models, which may not fully replicate the complex immune microenvironment of the human liver. This lack of humanized validation poses a key barrier to translation, as species-specific differences in chemokine signaling and immune cell function may interfere with predictions of clinical efficacy.

Among defined macrophage subsets, those expressing V-set and immunoglobulin domain containing 4 (VSIG4) have attracted particular attention due to their unique role in maintaining hepatic immune tolerance and suppressing excessive inflammation [10]. VSIG4, a single-pass transmembrane complement receptor mainly expressed on macrophages, functions as a critical immune checkpoint molecule and exhibits potent anti-inflammatory and tissue-protective properties in a variety of diseases [10–12]. Unlike broadly defined M2 macrophages, VSIG4^+^ macrophages (VSIG4^+^ Mφ) represent a naturally occurring, endogenous immunoregulatory subset with a well-defined molecular identity, offering a more precise and potentially safer therapeutic alternative. Although VSIG4 has been widely recognized as a classic marker of resident Kupffer cells (KCs), single-cell RNA-seq (scRNA-seq) analysis of ALI liver tissue and staining of our clinical ALI liver tissue samples both indicate that a subset of monocyte-derived macrophages (MoMFs) also upregulates VSIG4 expression in inflamed livers. We hypothesized that precise isolation of the VSIG4$^{+}$ MoMFs subset — rather than non-specific in vitro polarization — would more effectively recapitulate the intrinsic immunoregulatory functions required for ALI therapy. However, no study to date has explored VSIG4 $^{+}$ Mφ-based cell therapy, and whether ALI patients could benefit from such an approach remains unknown.

VSIG4^+^ Mφ represent a naturally occurring, endogenous immune regulatory subset with distinct molecular characteristics, offering a more precise and potentially safer therapeutic option. We hypothesize that infusing this specific subset can effectively alleviate ALI by restoring hepatic immune homeostasis. To test this, we employed a non-destructive immunomagnetic separation strategy to obtain VSIG4 $^{+}$ Mφ with high viability and evaluated their therapeutic efficacy in both a mouse APAP-induced ALI model and a vascularized human liver organoid system capable of reproducing key features of hepatocyte injury and monocyte chemotaxis. By providing cross-species evidence that therapeutic disruption of the CCL2-CCR2 chemotactic axis is feasible and effective, this study lays the preclinical foundation for advancing subset-specific macrophage immunotherapy toward clinical evaluation in ALI and other inflammation-driven liver diseases.

## Materials and methods

### Mouse model of APAP-induced ALI

Female C57BL/6 mice (8–10 weeks old, 20–25 g) were randomly assigned to three groups (*n* = 5 per group): (1) Control: intraperitoneal (i.p.) PBS; (2) ALI: i.p. APAP (250 mg/kg; Selleck, USA); (3) Treatment: i.p. APAP + tail vein injection of VSIG4$^{+}$ Mφ (1 × 10⁶ cells in 100 µL PBS) 12 h post-APAP. Mice were fasted overnight before APAP administration with free access to water. Forty-eight hours post-treatment, blood was collected for liver function tests, and livers were harvested for histology, RNA, and protein analysis. All animal experiments were approved by the Animal Experiments Ethical Committee of Tongji Hospital Affiliated to Tongji University.

### Cell culture and sample collection

The human monocytic cell line THP-1 (National Collection of Authenticated Cell Cultures, China) was cultured in RPMI-1640 medium (Gibco, USA) supplemented with 10% fetal bovine serum (FBS, Gibco, USA) and 1% sodium pyruvate. The mouse vascular endothelial cell line C166 (Yaji, China) and the mouse monocyte/macrophage cell line RAW264.7 (National Collection of Authenticated Cell Cultures, China) were cultured in DMEM containing 10% FBS. All cells were maintained at 37 °C in a humidified incubator with 5% CO₂. Liver tissue samples from healthy individuals and patients with ALI were obtained from the hepatobiliary surgery center of Tongji Hospital Affiliated to Tongji University (Table S1). The Tongji Hospital Affiliated to Tongji University Ethics Committee approved the use of human samples for this study, and informed consent was obtained from all the patients.

### Oligonucleotide design and synthesis

The oligonucleotide sequence was designed based on Endonuclease V (Endo V) cleavage specificity: 5’-ATGCGATCTdIGACTGAdITCGAATdICGGTACdICATGTA-3’. The 5’ end was modified with a carboxyl group (-COOH), and the 3’ end with an amino group (-Aminolinker C7). ‘dI’ denotes deoxyinosine cleavage sites. A control sequence lacking dI sites was also synthesized: 5’-ATGCGATCTAGACTGAATCGAATACGGTACACATGTA-3’. All oligonucleotides were synthesized by GENEWIZ (Suzhou, China).

### MNP@Oligo-VSIG4 conjugation and buffer optimization

Carboxylated magnetic nanoparticles (MNP) were activated with 10 mM EDC and 5 mM NHS in 0.1 M MES buffer (pH 4.5) for 15 min at room temperature with shaking (300 rpm). Activated MNP were incubated with animated oligonucleotide (20 µM final concentration) for 2 h at room temperature (200 rpm). After blocking with 1 mg/mL BSA, the MNP@Oligo conjugate was activated again with EDC/NHS and incubated with 0.1 mg anti-VSIG4 antibody (AntibodySystem SAS, France) for 2 h at room temperature. The final MNP@Oligo-VSIG4 conjugate was resuspended in PBS containing 1 mg/mL BSA and 0.05% NaN₃ and stored at 4$^{\circ}$C protected from light. The optimized isolation buffer (Buffer #2) contained: 10 mM HEPES (pH 7.4), 120 mM NaCl, 5 mM KCl, 5 mM glucose, 5 mM MgCl₂, and 0.5 mM reduced glutathione. Cell viability was assessed by trypan blue staining. Separation efficiency was determined by quantifying residual oligonucleotide after 15 min incubation with Endo V (1 U/µL; Haigene, China) at 37 °C.

### Scanning electron microscopy (SEM) sample preparation and imaging

The samples (MNP and MNP@Oligo-VSIG4) were naturally air-dried prior to analysis. SEM was performed following the guidelines of JY/T 0584–2020. Each sample was mounted onto conductive adhesive tape and sputter-coated with a conductive layer to enhance image quality. Imaging was conducted using a SEM (apreos 2c, Thermofisher, USA), and representative micrographs were captured at appropriate magnifications to assess surface morphology.

### Cell isolation using MNP@Oligo-VSIG4

Cells were resuspended in Buffer #2 at 1 × 10⁷ cells/mL and incubated with MNP@Oligo-VSIG4 (10 µL per 10⁷ cells) for 15 min at room temperature. After magnetic separation and washing, captured cells were incubated with Endo V (1 U/µL) in Buffer #2 for 15 min at 37 °C to release beads. Released cells were collected by centrifugation and used immediately for downstream applications. Purity was assessed by immunofluorescence, and viability was assessed by CCK-8 assay (Servicebio, China).

Bone marrow cells were flushed from femurs and tibias, subjected to red blood cell lysis, and cultured in RPMI-1640 with 10% FBS and 20 ng/mL M-CSF (Selleck, USA) for 5–7 days. Adherent cells were stimulated with C3b (10 µg/mL; Abclonal, China) for 24 h, then VSIG4^+^ cells were isolated using MNP@Oligo-VSIG4 as described above.

### Liver function tests

ALT or AST levels in serum and organoid culture supernatants were measured using the Amplex Red Alanine Aminotransferase Assay Kit or Amplex Red Aspartate Aminotransferase Activity Assay Kit (Beyotime, China), with procedures following the manufacturer’s instructions. Absorbance at 570 nm was measured using a microplate reader (ThermoFisher, USA), and transaminase activity was calculated based on a standard curve.

### Histology and immunohistochemistry (IHC)

Liver tissues were fixed in 4% paraformaldehyde (PFA), paraffin-embedded, and sectioned (4–5 μm). Hematoxylin and eosin (H&E) staining was performed for morphological assessment. For IHC, sections were incubated with primary antibodies against F4/80 (Servicebio, China) or MPO (Servicebio, China) followed by HRP-conjugated secondary antibodies and DAB development (Table S2).

### TUNEL assay

Apoptosis was detected using the Tunel Cell Apoptosis Detection Kit (Servicebio, China) according to the manufacturer’s instructions. Apoptotic cells (green fluorescence) and DAPI-stained nuclei (blue) were visualized by fluorescence microscopy Pannoramic MIDI‌ (3D HISTECH, Hungary).

### Immunofluorescence staining

Frozen liver tissue or organoid sections, or cultured cells were fixed with 4% PFA, permeabilized with 0.3% Triton X-100, and blocked with 10% horse serum. Primary antibodies against VSIG4 (Abmart, China), CCR2 (Servicebio, China), F4/80 (Servicebio, China), CD31 (Abmart, China), HNF4α (Proteintech, China), α-SMA (Proteintech, China), or CD68 (Huilan biotech, China) were incubated overnight at 4$^{\circ}$C, followed by Alexa Fluor-conjugated secondary antibodies (Table S2). Sections were mounted with DAPI-containing mounting medium and imaged using the Pannoramic MIDI‌ (3D HISTECH, Hungary) and analyzed using HLB-viewer software.

### Flow cytometry

Cells were fixed with 4% PFA, blocked with goat serum, and incubated with antibodies against VSIG4 (Abmart, China) and CCR2 (Servicebio, China) for 1 h at room temperature, followed by Alexa Fluor 488- or 568-conjugated secondary antibodies (Table S2). Data were acquired on a CytoFLEX S flow cytometer (Beckman, USA) and analyzed using CytExpert software. Single-color stained tubes served as fluorescence-minus-one (FMO) controls for gating: CCR2-only as VSIG4 FMO, and VSIG4-only as CCR2 FMO. Isotype controls were used to confirm specificity.

### Quantitative real-time PCR

Total RNA was extracted using SteadyPure Rapid RNA Extraction Kit (Accurate biology, China), and cDNA was synthesized using PrimeScript™ RT Master Mix (Takara, Japan). qPCR was performed using TB Green Premix Ex Taq™ II (Takara, Japan) on a SLAN-96 S PCR Analysis System (Zeesan, China). Primer sequences are listed in Table S3. Relative expression was calculated using the 2^-ΔΔCt^ method with β-actin as the internal control.

### Immunoblotting

Total protein was extracted using RIPA buffer (Beyotime, China) containing protease and phosphatase inhibitors. Proteins were separated by SDS-PAGE, transferred to PVDF membranes, and incubated with primary antibodies against Bcl-2 (Abmart, China), Bax (Selleck, USA), phospho-p65 (Abmart, China), CCL2 (Abmart, China), and β-actin (Servicebio, China) (Table S2). HRP-conjugated secondary antibodies were detected using ECL Basic Femto Kit (Abclonal, China).

### Human liver organoid culture and characterization

Human healthy liver tissue was obtained from surgical resections with informed consent. Tissue was washed 3–5 times with organoid washing solution (Lisheng Biotech, China), and minced into 1–2 mm³ pieces and cultured in 6 cm dish (Corning, USA) with Liver Organoid Culture Medium (Lisheng Biotech, China). Organoids were cultured at 37$^{\circ}$C with 5% CO₂, with half of the medium changes every 3–5 days. After 7–10 days, organoids were characterized by immunofluorescence for HNF4α, CD31, α-SMA, and CD68 (Table S2).

### Organoid ALI model and co-culture

For organoid ALI model, mature organoids were treated with APAP (2–20 mM) for 2–12 h. Supernatants were collected for ALT/AST and LDH assays. For co-culture experiments, organoids were placed in the lower chamber of Transwell plates (8.0 μm pore size; Beyotime, China), and THP-1 cells (1 × 10⁵) were added to the upper chamber with or without APAP (2 mM). After 12 h, organoids and cells were collected for qPCR, immunoblotting, and immunofluorescence analysis. LDH was detected using the LDH Cytotoxicity Assay Kit (Beyotime, China) according to the manufacturer’s instructions. Measure the absorbance at 450 nm using a microplate reader (ThermoFisher, USA), with 650 nm as the reference wavelength, and calculate the LDH levels based on the standard curve.

### RNA sequencing (RNA-seq) and data analysis

Total RNA was extracted from each sample, and its quality was assessed. mRNA was enriched using Oligo(dT) magnetic beads and fragmented into short fragments. The cDNA libraries were sequenced on the Illumina sequencing platform by Metware Biotechnology Co., Ltd. (Wuhan, China). Raw reads were filtered using fastp to remove adapter sequences, reads with more than 10% ambiguous bases, or reads with over 50% low-quality bases (Q ≤ 20). Clean reads were aligned to the mouse reference genome. Gene expression levels were quantified as FPKM with featureCounts. Differentially expressed genes (DEGs) between groups were identified using DESeq2, with a threshold of log2 fold change ≥ 1 and false discovery rate < 0.05.

### Single-cell RNA sequencing data analysis

Public scRNA-seq datasets (GSE192742, GSE280515 and GSE280514) were downloaded from GEO, and analyzed based on Liver Cell Atlas (https://www.livercellatlas.org/index.php). Demultiplexing of the raw data was performed using the CellRanger software. The read sequences obtained after demultiplexing are used as input for “cellranger count” (CellRanger software). This tool uses STAR to align the read sequences to the reference genome and aggregates them into unique molecular identifier (UMI) counts. The final output is a large numerical expression matrix, where rows represent cell barcodes and columns represent gene identifiers. Aggregate analysis of the samples is performed using “cellranger aggr” (CellRanger software).

### Statistical analysis

Data are presented as mean ± SEM. Comparisons between two groups were performed using unpaired two-tailed Student’s t-test. Multiple group comparisons were analyzed by one-way ANOVA followed by Tukey’s multiple comparisons test. Correlations were assessed by Pearson correlation coefficient. *P* < 0.05 was considered statistically significant. Analyses were performed using GraphPad Prism 9.0, Image J, R software and Origin 2024.

## Results

### VSIG4^+^ kupffer cells are depleted during ALI, coinciding with pro-inflammatory microenvironment establishment

**Fig. 1:**
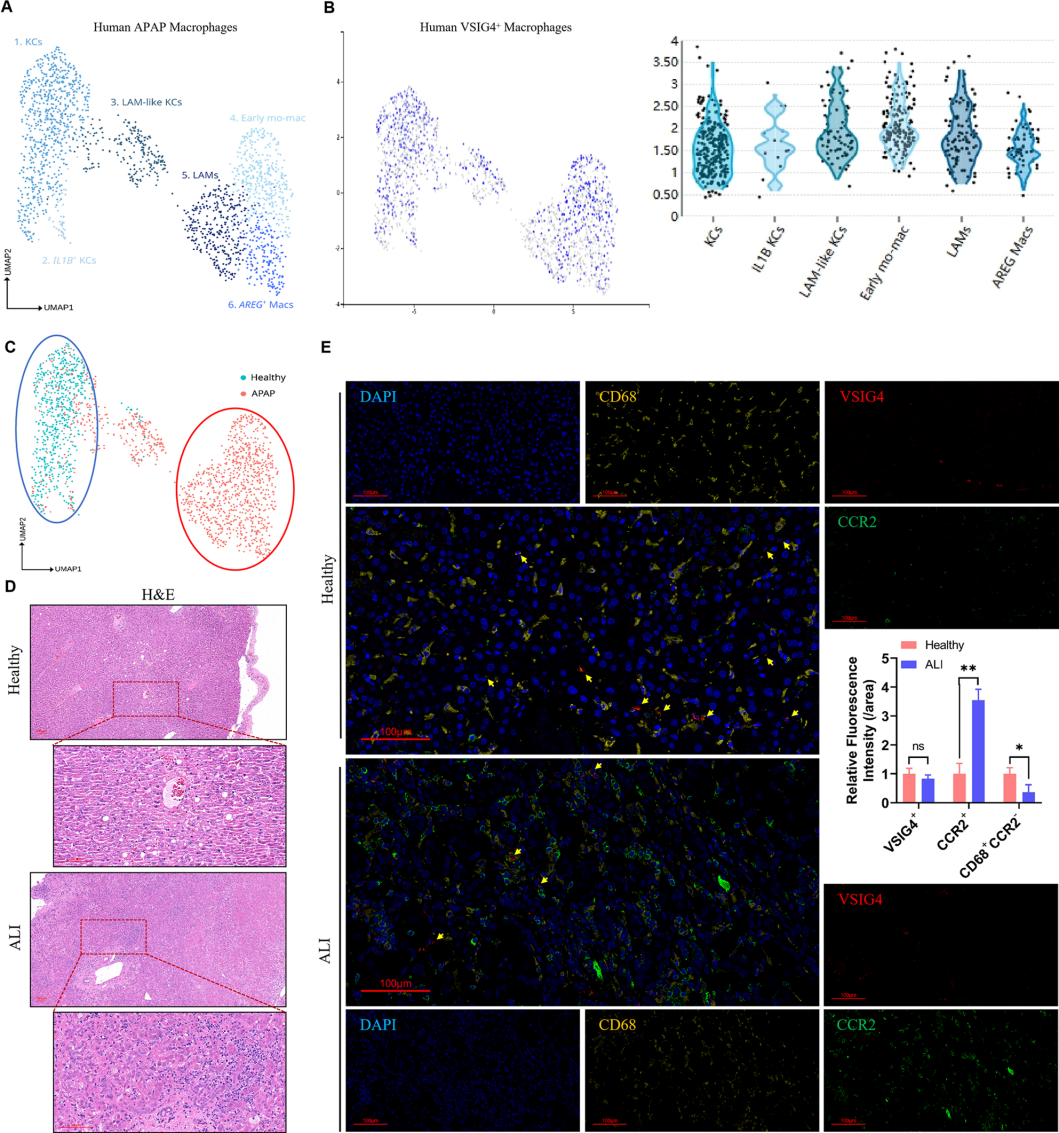
Analysis of scRNA-seq data on changes in hepatic macrophage populations in ALI patients and clinical sample testing. **(A)** The annotated UMAP highlighted six distinct clusters formed in human APAP macrophage. **(B)** Left: UMAP plot showing VSIG4^+^ cells (blue, UMI > 0) and negative cells (grey). Right: Violin plots of expression levels (log-normalized) in VSIG4^+^ cells across clusters. Violin width indicates kernel density; dots represent individual cells. **(C)** Group human macrophage UMAP plots by healthy individuals (healthy) and ALI (APAP), and compare the changes in the distribution of VSIG4^+^ macrophages. **(D)** H&E staining of liver tissues of healthy (*n* = 3) and ALI patients (*n* = 3). Scale bars, 100 μm. **(E)** Immunofluorescence staining and statistical results for markers of various cell types (CD68: marker of hepatic macrophages; CCR2: marker of MoMFs, and VSIG4) in liver tissue from healthy (*n* = 3) and ALI patients (*n* = 3). Scale bar: 100 μm. *p < 0.05, **p < 0.01, ns: no significant difference

To delineate macrophage dynamics during ALI, we first interrogated public and internal scRNA-seq datasets from human and murine livers. Analysis of human liver scRNA-seq data revealed that VSIG4 is primarily expressed in MoMFs and KCs (Supplementary Fig. 1). KCs were significantly reduced in the livers of patients with APAP-induced ALI compared to healthy individuals, and early MoMFs, lipid-associated macrophages (LAMs), and AREG^+^ macrophages (all originate from monocytes) were significantly expanded (Fig. 1A-C). In samples from ALI patients, histological analysis verified significant liver injury and immune cell infiltration (Fig. 1D). Immunofluorescence staining revealed increased MoMFs (CCR2^+^) and decreased KCs (CD68^+^CCR2^-^) in ALI livers compared to healthy controls (Fig. 1E). Although total VSIG4^+^ Mφ numbers did not change dramatically due to infiltrating MoMFs (including some VSIG4^+^ MoMFs), the presence of VSIG4^+^ MoMFs (CCR2^+^VSIG4^+^) within ALI patient livers was confirmed (Fig. 1E).

**Fig. 2:**
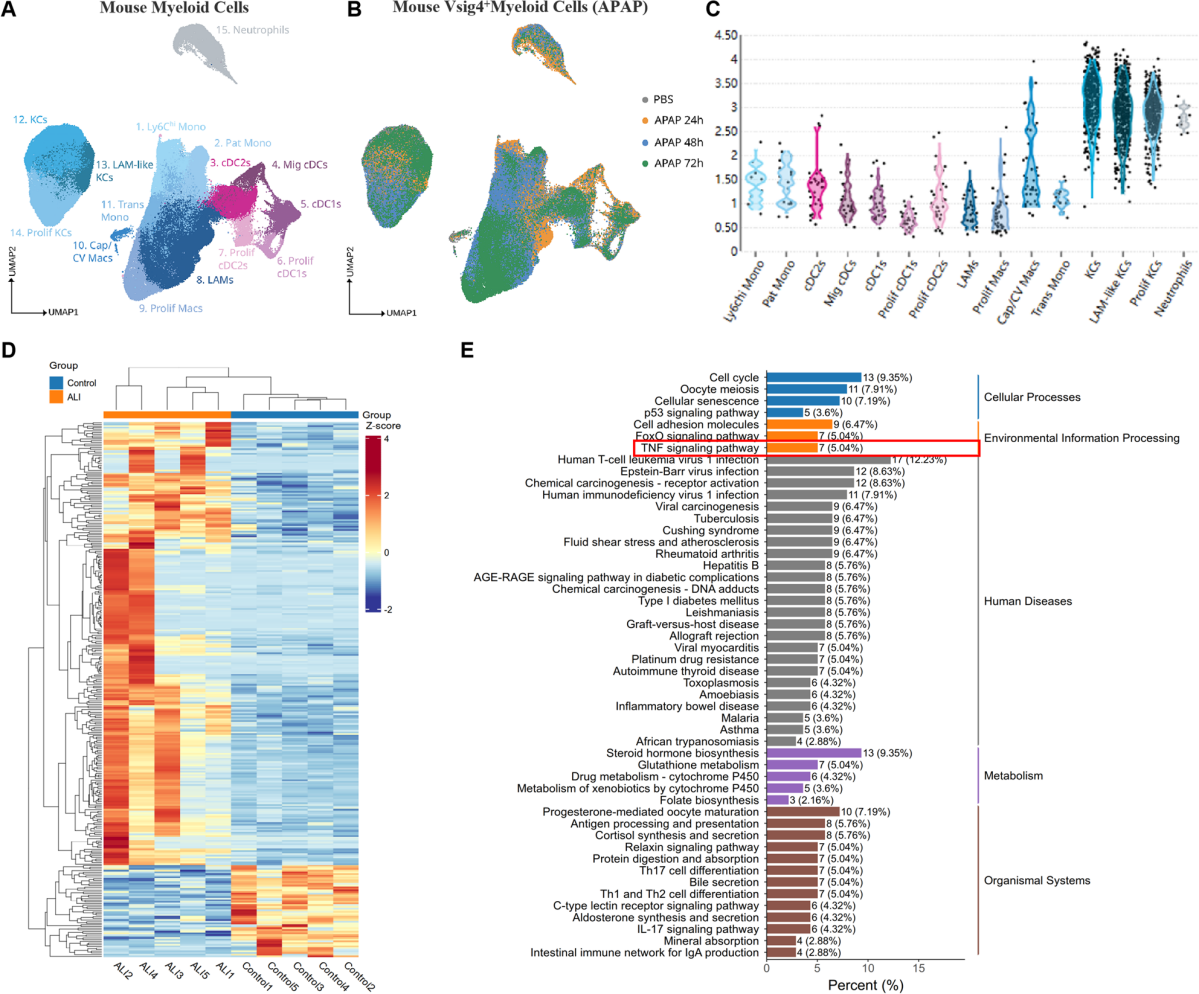
Analysis of scRNA-seq data and RNA-seq revealing changes in hepatic macrophage populations and the progression of inflammation in a mouse ALI model. **(A)** The annotated UMAP highlighted fifteen distinct clusters formed in mouse myeloid cells. **(B)** Group mouse myeloid cells UMAP plots by different APAP treatment time, and compare the changes in the distribution of Vsig4^+^ myeloid cells. **(C)** Display the UMAP plot of mouse myeloid cells stratified by VSIG4 expression to highlight the distribution of VSIG4^+^ myeloid cells. **(D)** Heatmap of DEGs from RNA-seq of mouse liver tissues (APAP-ALI vs. control) (*n* = 5 for each group). **(E)** The KEGG enrichment barplot enriched among the DEGs. “Organismal systems” refers to one of the KEGG category encompassing pathways such as immune system and endocrine system. The red box highlights the key pathways associated with the inflammatory microenvironment during ALI

Murine Vsig4 expression was similarly enriched in MoMFs and KCs, which is consistent with human findings (Supplementary Fig. 2). Dynamic changes in hepatic macrophage subpopulations were found by analyzing scRNA-seq data from an APAP-induced ALI mouse model. While proliferating KC and LAM-like KC subclusters significantly increased at 48–72 h, probably indicating the repair stage, LAM-like KC subcluster expanded at 24 h post-APAP (Fig. 2A-C). To specifically assess Vsig4 dynamics during ALI, we established APAP-induced ALI mouse models. RNA-seq analysis revealed that, compared with the control group, the expression of genes such as Tnf, Ccl2, Ccl22, Cxcl2, and Ly6c2 was significantly elevated in ALI group, reflecting further exacerbation of myeloid cell chemotaxis and inflammatory responses following the depletion of hepatic KCs (Fig. 2D; Tables S4). Functional enrichment analysis revealed abnormal activation of pathways related to metabolism, chemokine signaling, and TNF signaling (Fig. 2E; Tables S5).

Collectively, these findings reveal that ALI progression is characterized by depletion of resident macrophages (numerous VSIG4^+^ KCs) and establishment of an overactivated inflammatory microenvironment. Despite infiltration by MoMFs, the proportion of VSIG4^+^ MoMFs remains low. Conversely, extensive infiltration of immune cells leads to an overactivated inflammatory response and secondary liver injury, which is a key factor in the progression of ALI to ALF. VSIG4 is presumed to exert anti-inflammatory and pro-reparative effects during ALI [13]. These prompted us to speculate that exogenous VSIG4$^{+}$ Mφ supplementation could improve immunoregulatory function and mitigate ALI progression. However, to achieve this therapeutic goal, it would be optimal to develop a non-destructive method to obtain sufficient numbers of viable and functionally intact VSIG4$^{+}$ Mφ.

### Development and validation of a reversible immunomagnetic platform for non-destructive isolation of viable VSIG4^+^ Mφ

**Fig. 3:**
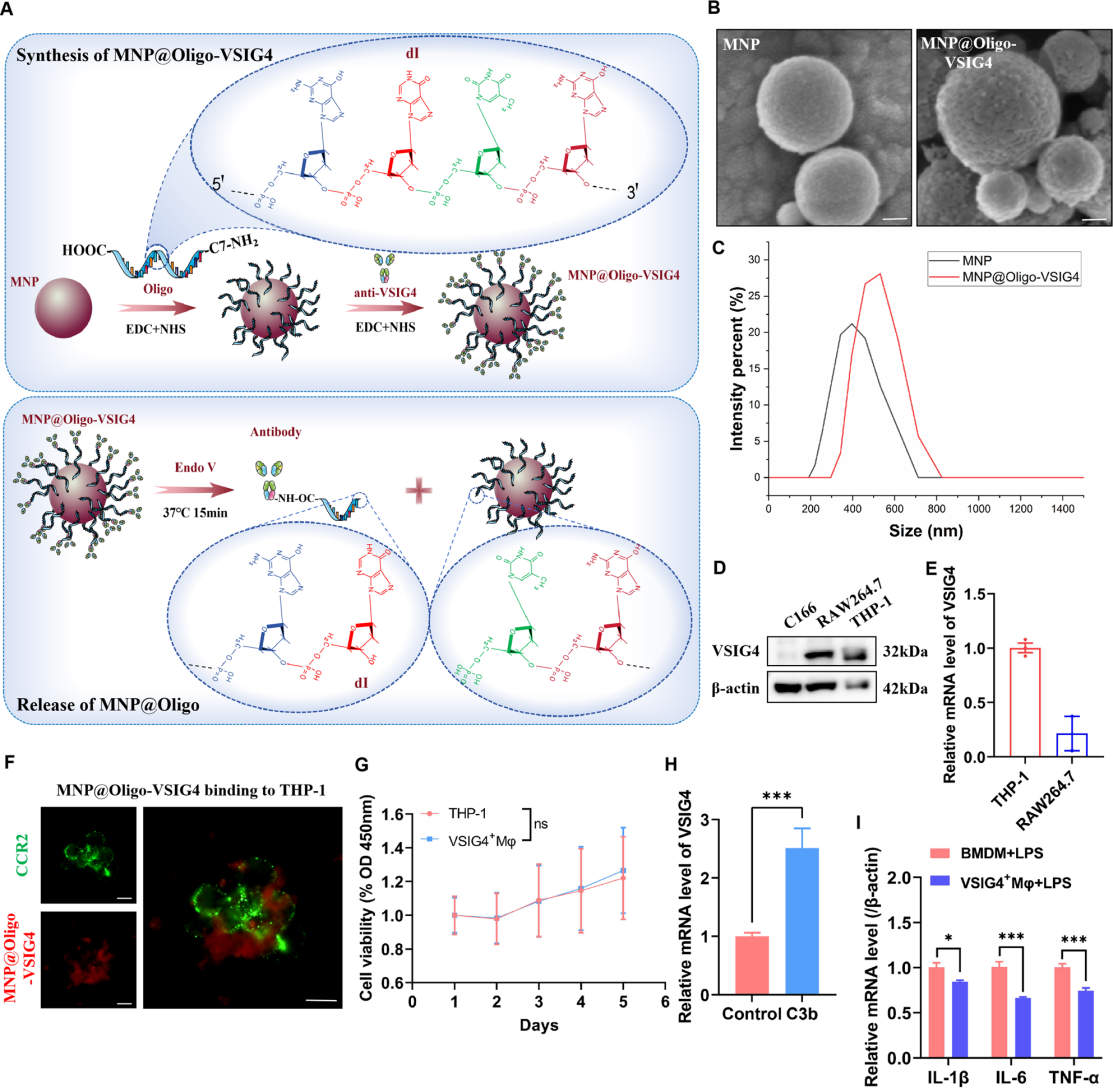
Design and characterization of reversible immune-affinity MNP@Oligo-VSIG4 nanoparticles. **(A)** Schematic illustration of the construction of MNP@Oligo-VSIG4. Iron oxide magnetic nanoparticles (MNPs) were surface-functionalized with oligonucleotide-conjugated anti-VSIG4 aptamers. The scheme also depicts the enzymatic cleavage strategy used to release the aptamer-bound cells. **(B)** Representative scanning electron microscopy (SEM) images of unmodified MNP and MNP@Oligo-VSIG4. Scale bar, 200 nm. **(C)** Hydrodynamic diameters (Z-average) of MNP and MNP@Oligo-VSIG4 measured by dynamic light scattering (DLS). **(D)** VSIG4 protein expression levels in the human monocytic cell line THP-1, the mouse macrophage cell line RAW264.7, and the mouse endothelial cell line C166, assessed by Western blot. β-actin was used as a loading control (*n* = 3). **(E)** Relative VSIG4 mRNA levels in THP-1 and RAW264.7 cells determined by RT-qPCR. Data are normalized to β-actin (*n* = 3). **(F)** Representative immunofluorescence images demonstrating the binding of MNP@Oligo-VSIG4 (red) to THP-1 cells (CCR2, green) (*n* = 3). Scale bar, 50 μm. **(G)** Viability of THP-1 cells before and after magnetic separation with MNP@Oligo-VSIG4, assessed by CCK-8 assay (*n* = 3). **(H)** RT-qPCR analysis of VSIG4 mRNA levels in THP-1 cells following stimulation with complement C3b for 12 h. Data are normalized to β-actin and expressed as fold change relative to the unstimulated control (0 h) (*n* = 3). **(I)** RT-qPCR analysis of IL-1β, IL-6 and TNF-α mRNA levels in BMDM before and after MNP@Oligo-VSIG4 separation following stimulation with LPS for 12 h (*n* = 5). *p < 0.05, ***p < 0.001, ns: no significant difference

Conventional immunomagnetic beads permit target cell capture but not subsequent bead detachment, which could compromise cell viability and regulatory compliance for therapeutic applications [14, 15]. To facilitate non-destructive isolation of VSIG4^+^ Mφ appropriate for cell therapy, we developed a reversible immunomagnetic platform (Fig. 3A). Magnetic nanoparticles (MNPs) were conjugated with an anti-VSIG4 antibody via an oligonucleotide linker containing Endonuclease V (Endo V)-cleavable deoxyinosine (dI) sites. The linker was efficiently cleaved by Endo V within 30 min, allowing for the gentle detachment of bound cells from the magnetic beads (Supplementary Fig. 3A-D). Electron microscopy showed surface roughening following conjugation (Fig. 3B and Supplementary Fig. 3E), and increased hydrodynamic diameter was confirmed by dynamic light scattering (Fig. 3C). The successful conjugation of the oligonucleotide and antibody was confirmed by molecular characterization (Supplementary Fig. 3F and G). All isolation procedures were performed in an optimized buffer that maintains both high cell viability and efficient enzymatic cleavage (Supplementary Fig. 4A-D).

To validate isolation efficiency, we utilized THP-1 cells (VSIG4^+^CCR2^+^) as positive targets and C166 cells (VSIG4^-^) as negative background (Fig. 3D and E).Using this system, VSIG4^+^ cells were captured with > 80% efficiency and subsequently released with > 95% efficiency following enzymatic cleavage (Supplementary Fig. 4E-G). Direct visualization confirmed the specific binding of MNP@Oligo-VSIG4 to the surface of VSIG4^+^ THP-1 cells (Fig. 3F). This isolation procedure did not compromise cell viability, with post-isolation viability remaining above 95% (Fig. 3G). This platform thus provides a rapid, efficient, and non-cytotoxic method for obtaining highly pure and viable VSIG4^+^ macrophages for downstream therapeutic application. Notably, stimulation with the VSIG4 ligand C3b significantly increased VSIG4 expression (Fig. 3H), and under LPS challenge, VSIG4^+^ bone marrow-derived monocytes (BMDM) produced markedly lower levels of pro-inflammatory cytokines (IL-1β, IL-6, TNF-α) compared with unselected BMDMs (Fig. 3I), indicating a more stable anti-inflammatory phenotype.

### VSIG4^+^ Mφ ameliorate liver injury in APAP-induced ALI

**Fig. 4:**
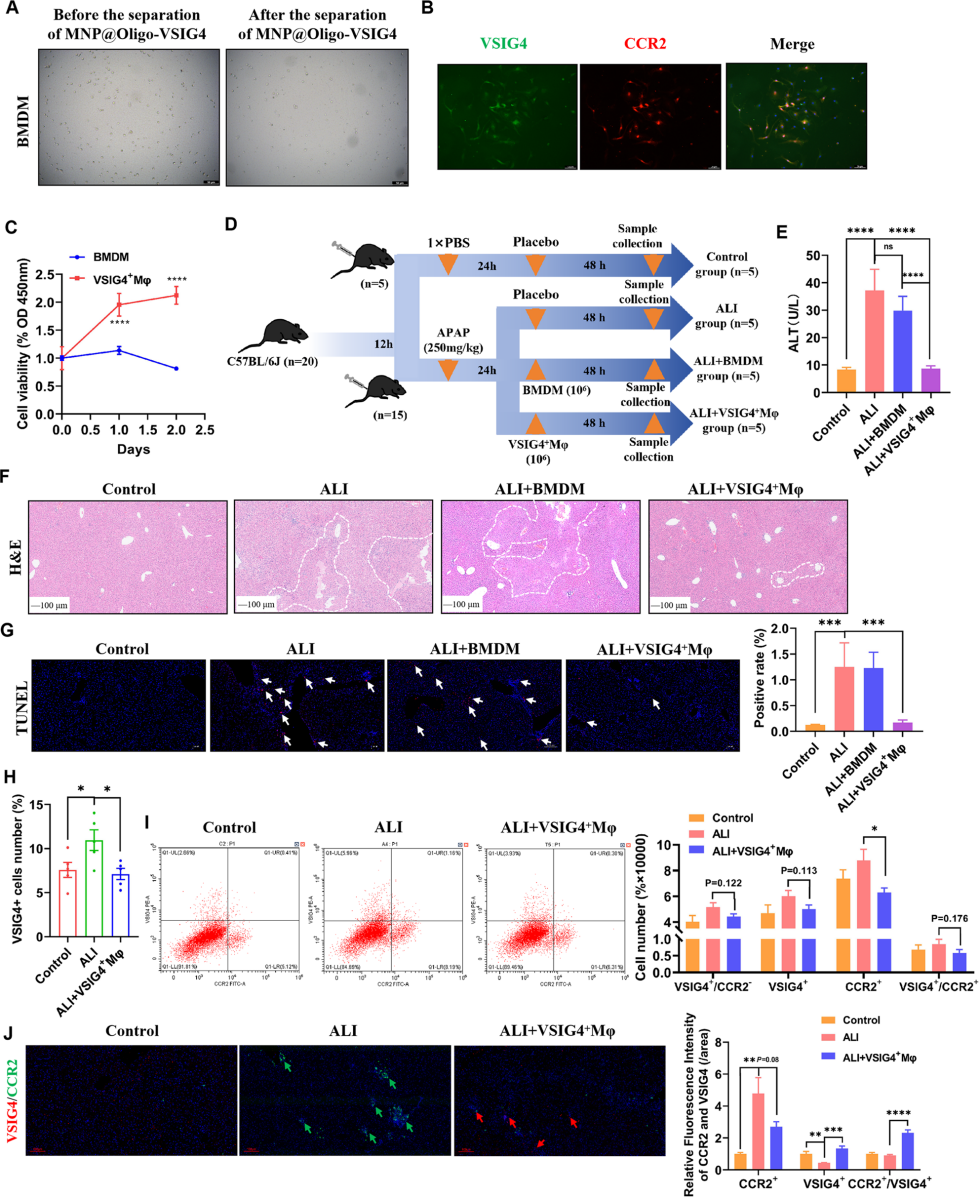
VSIG4^+^ MoMFs ameliorate liver injury in APAP-induced ALI. **(A)** Comparison of the morphology of BMDM before and after MNP@Oligo-VSIG4 separation (*n* = 3). **(B)** Immunofluorescence staining of VSIG4 and CCR2 in bone marrow-derived macrophages (*n* = 3). Scale bar: 50 μm. **(C)** Comparison of BMDM cell viability before and after MNP@Oligo-VSIG4 separation (*n* = 3). **(D)** Schematic diagram illustrating the protocol and grouping for ALI modeling and BMDM or VSIG4^+^ macrophages (VSIG4^+^ Mφ) cell therapy in mice (*n* = 5 for each group). **(E)** Comparison of ALT levels among mice in each group (*n* = 5). **(F)** Representative H&E staining images of mice livers in each group. The white dashed lines indicate the necrotic areas (*n* = 5). Scale bar: 100 μm. **(G)** Representative TUNEL staining images of mice livers in each group and quantitative analysis of TUNEL^+^ cells (*n* = 5). Scale bar: 100 μm. **(H)** Comparison of the percentage of VSIG4^+^ cells among total myeloid mononuclear cells (VSIG4^+^ cells/total myeloid mononuclear cells×100%) from each group of mice using MNP@Oligo-VSIG4 (*n* = 5). **(I)** Flow cytometry was used to compare the abundance of specific cell populations in bone marrow-derived cells from each group of mice (*n* = 5). **(J)** Representative immunofluorescence staining images of liver tissue from mice in each group, along with statistical results regarding the number of specific cell populations (*n* = 5). Scale bar: 100 μm. *p < 0.05, **p < 0.01, ***p < 0.001, ****p < 0.0001

Based on scRNA-seq data showing that VSIG4 is expressed in MoMFs (Supplementary Fig. 2), we isolated mouse BMDM, induced their differentiation into macrophages using M-CSF, and stimulated them with C3b to enhance VSIG4 expression. Using the MNP@Oligo-VSIG4 platform, we purified high-purity, high-viability VSIG4^+^ MoMFs (Fig. 4A-C). Immunofluorescence confirmed that these isolated cells mostly co-express VSIG4 and CCR2 (Fig. 4B), suggesting their ability to respond to CCL2 gradients and chemotax toward damaged liver tissue. In an APAP-induced ALI mouse model (Fig. 4D), tail vein injection of VSIG4^+^ Mφ (1 × 10^6^ cells) significantly decreased serum ALT levels compared to placebo group and BMDM gorup (equal amount of unselected BMDM was injected) (Fig. 4E). Following VSIG4^+^ Mφ transfer, histological analysis and TUNEL staining showed a significant decrease in APAP-induced necrosis and apoptosis (Fig. 4F and G).

Quantification of bone marrow VSIG4^+^ cells using MNP@Oligo-VSIG4 revealed increased VSIG4^+^ cells in ALI mice compared to controls, but this number was substantially reduced in VSIG4^+^ Mφ-treated mice (Fig. 4H). Flow cytometry confirmed parallel changes in VSIG4^+^ and CCR2^+^ cells (Fig. 4I, Supplementary Fig. 5). Moreover, we analyzed other two populations — VSIG4^+^/CCR2^−^ and VSIG4^+^/CCR2^+^ cells — to distinguish between recruited MoMFs (VSIG4^+^/CCR2^+^) and cells that may have down-regulated CCR2 during anti-inflammatory reprogramming or that represent a resident/precursor pool (VSIG4^+^/CCR2^−^). This stratification helps infer the inflammatory status of the VSIG4^+^ compartment during ALI (Fig. 4I). However, immunofluorescence of liver tissue showed decreased intrahepatic VSIG4^+^ cells (reflecting KCs depletion) but increased CCR2^+^ cells in ALI mice. Conversely, VSIG4^+^ Mφ treatment increased both VSIG4^+^ and CCR2^+^ cells within the liver (Fig. 4J). These findings imply that adoptively transferred VSIG4^+^ Mφ engraft in the liver, possibly neutralizing or inhibiting chemotactic signals and reducing demand for bone marrow-derived CCR2^+^ cell mobilization—consistent with the observed decline in bone marrow CCR2^+^ cells (Fig. 4J).

### VSIG4^+^ Mφ suppress hepatic inflammation by inhibiting CCL2-mediated immune cells recruitment

**Fig. 5:**
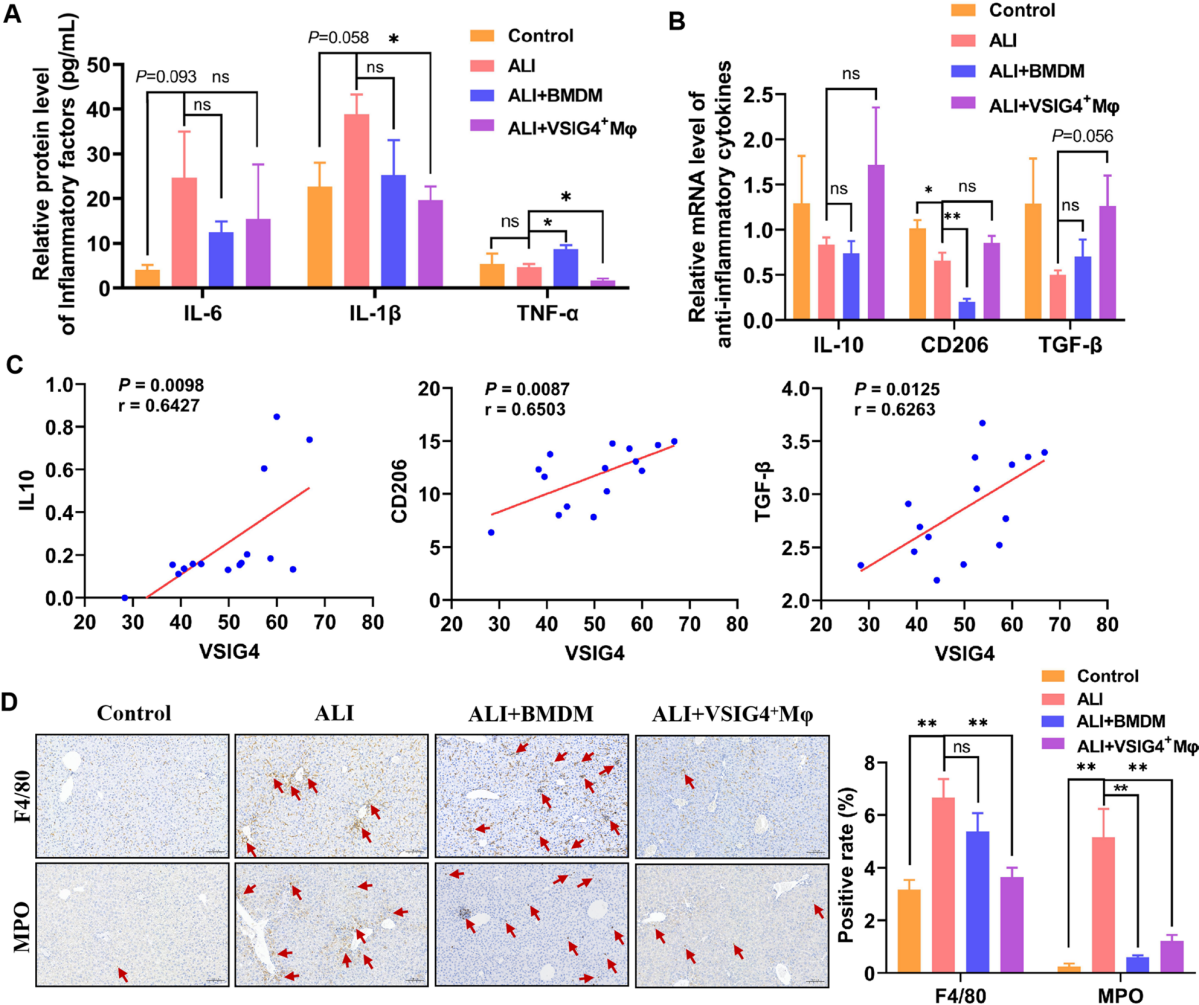
VSIG4^+^ MoMFs suppress hepatic inflammation. **(A)** The secretion levels of inflammatory factors (IL-1β, IL-6, and TNF-α) in the serum of mice in each group (*n* = 5). **(B)** The relative mRNA expression levels of genes related to anti-inflammation (IL-10, CD206, and TGF-β) in the liver tissues of mice in each group (*n* = 5). **(C)** Analysis of the correlation between the mRNA expression of anti-inflammatory factors and VSIG4 expression in the liver tissues of mice in each group (*n* = 15). **(D)** Representative liver staining images with quantification of F4/80 and MPO in each group (*n* = 5). Scale bar: 100 μm. *p < 0.05, **p < 0.01

Next, we investigated the mechanism underlying VSIG4^+^ Mφ-mediated protection. Treatment with VSIG4^+^ macrophages dramatically decreased intrahepatic pro-inflammatory cytokines (IL-1β, IL-6, TNF-α) while increasing anti-inflammatory mediators (IL-10, TGF-β1) and the M2 macrophage marker MRC1 (CD206), whereas unselected BMDM not only failed to recapitulate these effects but also induced opposite changes in TNF-α and CD206 (Fig. 5A, B; Supplementary Fig. 6A). Anti-inflammatory cytokine levels positively correlated with VSIG4 expression, whereas pro-inflammatory cytokines showed no correlation (Fig. 5C; Supplementary Fig. 6B), indicating that VSIG4^+^ Mφ primarily performs anti-inflammatory functions. Reduced immune cell infiltration in treated livers was verified by histochemical analysis (Fig. 5D).

**Fig. 6:**
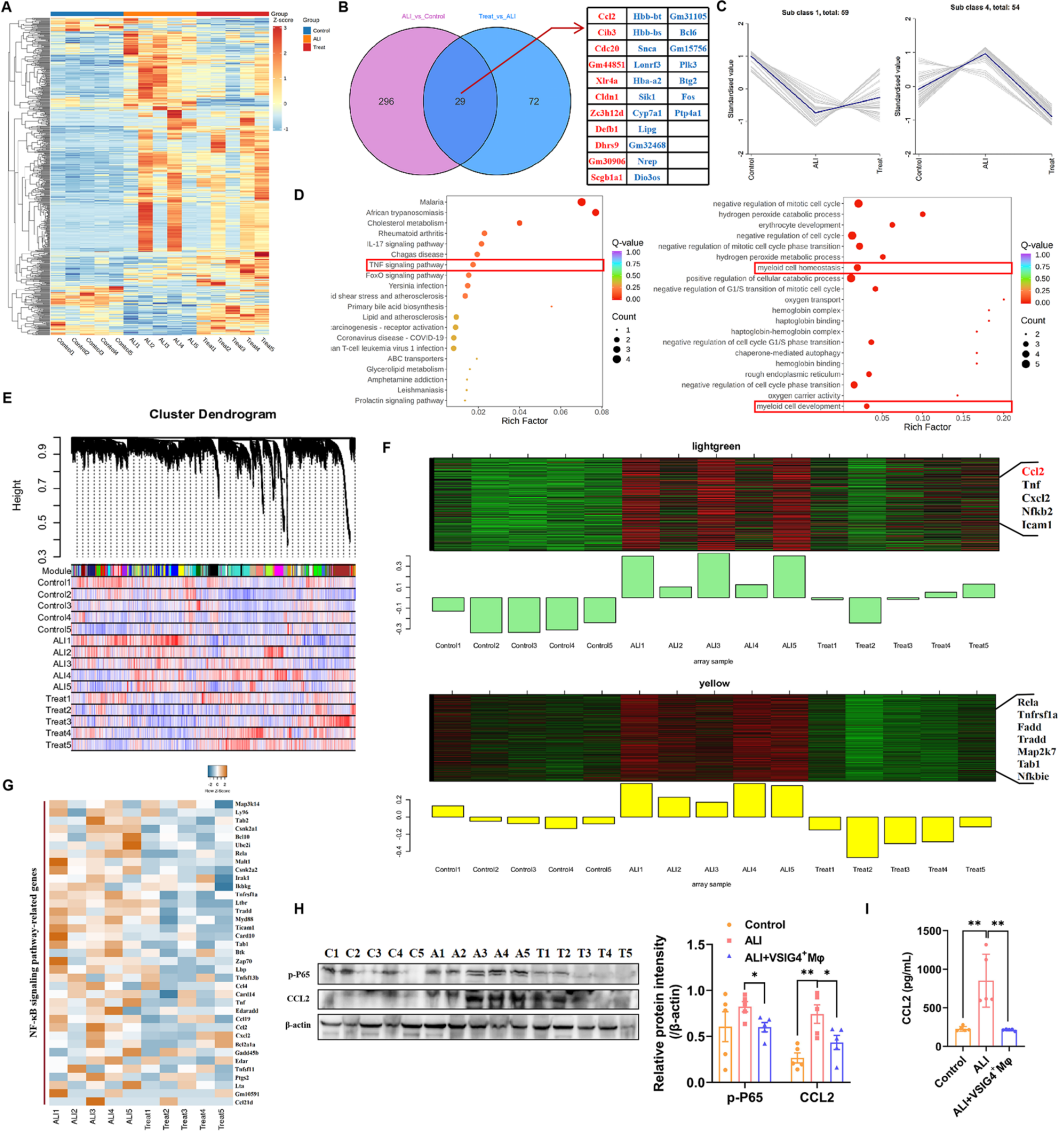
VSIG4^+^ MoMFs inhibit CCL2-mediated immune cells recruitment by suppressing the NF-κB pathway. **(A)** Heatmap of DEGs in ALI group compared with control and VSIG4^+^ MoMFs treatment compared ALI group (*n* = 5 for each group). **(B)** Combined analysis of the Venn diagrams and gene lists showing differentially expressed genes across the three groups. **(C)** K-means cluster analysis shows that genes within the same class exhibit similar expression trends across different experimental treatments. Subclass 1: represents the cluster of genes with downregulated expression in ALI group; Subclass 4: represents the cluster of genes with upregulated expression in ALI group. (*n* = 5) **(D)** The KEGG and GO enrichment barplot enriched among the 29 DEGs. The red box highlights the key pathways associated with the inflammatory microenvironment and myeloid cell homeostasis and development pathways during ALI (*n* = 5). **(E)** The WGCNA analysis displays a heatmap of gene clustering across modules, reflecting the overall expression patterns of genes across samples and modules (*n* = 5). **(F)** The WGCNA analysis examines the expression patterns of the lightgreen and yellow modules across the three groups, as well as the expression levels of the inflammation-, chemotaxis-, and NF-κB-related genes contained within them (*n* = 5). **(G)** Heatmap show the intrahepatic expression of members of NF-κB pathways (*n* = 5). **(H)** Immunoblotting analysis and quantification of p-P65 and CCL2 expression in liver tissues from mice in each group (*n* = 5). **(I)** The secretion levels of CCL2 in the serum of mice (*n* = 5). *p < 0.05, **p < 0.01

According to RNA-seq analysis, VSIG4^+^ Mφ treatment reversed 29 differentially expressed genes (Fig. 6A-C; Tables S6), with functional enrichment in pathways associated with inflammatory mediator secretion, immune cell chemotaxis, and myeloid cell homeostasis and development pathways (Fig. 6D; Supplementary Fig. 7; Tables S7-9). Interestingly, Ccl2, which codes for the important monocyte chemoattractant MCP-1, was upregulated in ALI but downregulated after treatment with VSIG4^+^ Mφ (Fig. 6A-C). The CCL2-CCR2 axis is central to myeloid cell recruitment during inflammatory amplification [16, 17]. This not only reaffirms our previous findings that VSIG4^+^ Mφ alleviate ALI by improving the liver inflammatory microenvironment, but also suggests that VSIG4^+^ Mφ may mitigate secondary inflammatory damage by blocking CCL2-CCR2 axis-dependent chemotactic recruitment of myeloid monocytes.

The primary transcription factor signaling pathways mediating CCL2 transcription and expression during ALI are mainly NF-κB pathway [18]. Consistent with these findings, WGCNA analysis performed cross-module gene clustering, and the results showed that the light green and yellow modules, which contain genes associated with inflammation, chemotaxis, and NF-κB, exhibited expression patterns consistent with those of CCL2 across the three subgroups, decreasing following treatment with VSIG4^+^ Mφ (Fig. 6E, F). Expression of NF-κB target genes was elevated in ALI and suppressed by VSIG4^+^ Mφ treatment (Fig. 6G). At the protein level, we also demonstrated that VSIG4^+^ Mφ treatment significantly suppressed phosphorylation of NF-κB p65, a master transcriptional regulator of CCL2. This leads to a significant reduction in CCL2 expression (Fig. 6H, I), which in turn diminishes the chemotactic gradient responsible for the recruitment of CCR2^+^ pro-inflammatory monocytes and macrophages into the injured liver. Previous research has demonstrated that VSIG4 inhibits NF-κB-mediated inflammation in myocardial ischemia and metabolic liver disease [19, 20]. These results confirm and expand upon these findings by showing that VSIG4^+^ Mφ inhibits the NF-κB-CCL2 axis, preventing inflammatory amplification caused by CCR2^+^ monocyte/macrophage recruitment.

### Generation of human liver organoids that recapitulate the multicellular hepatic microenvironment

**Fig. 7:**
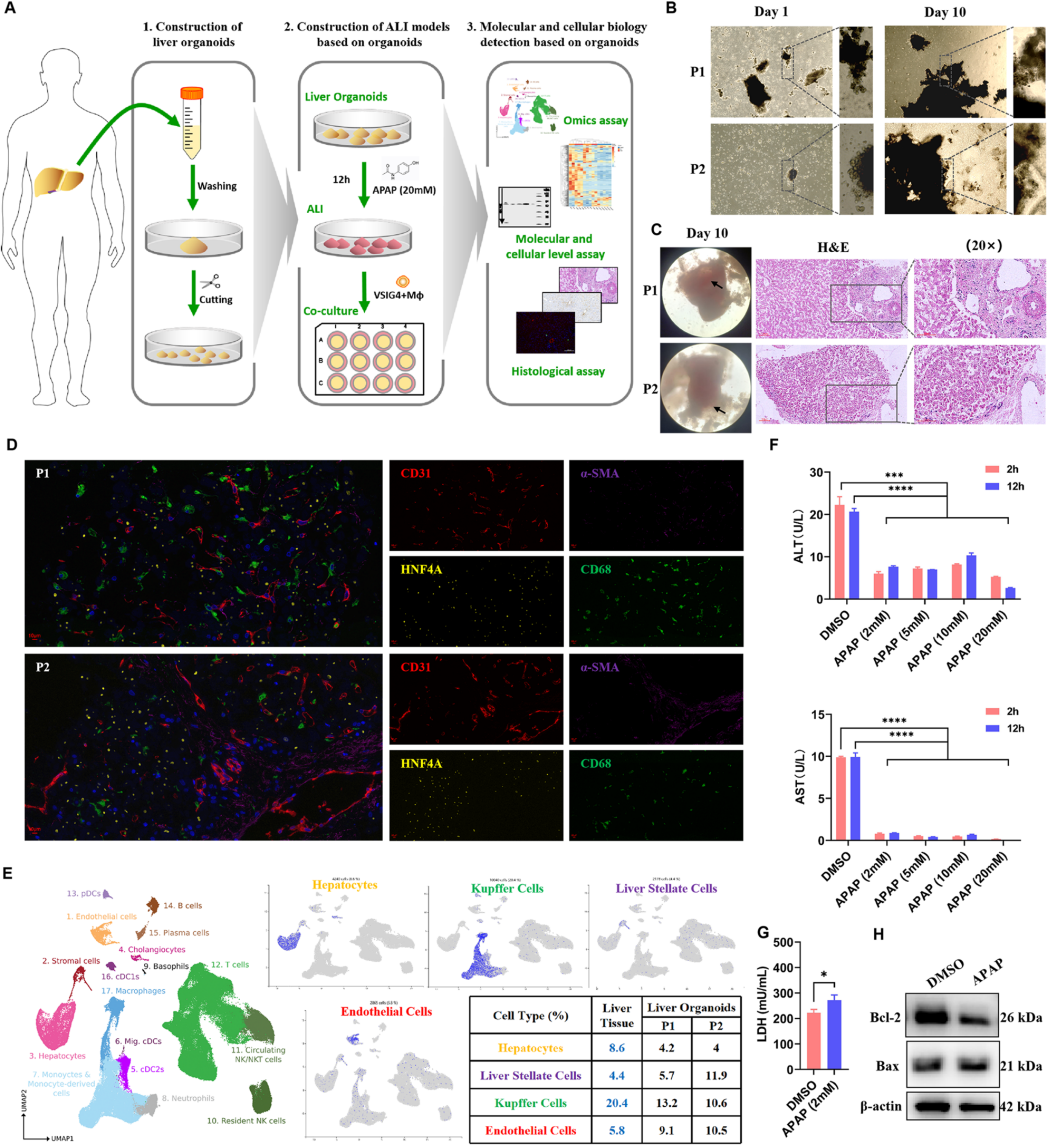
Construction and characterization of human liver organoids and ALI models based on liver organoids. **(A)** Schematic diagram of the workflow for constructing human-derived normal liver organoids and the resulting ALI and chemotaxis models. **(B)** Representative bright-field images of human liver organoids derived from two donors (P1 and P2), showing growth from day 1 to day 10 (*n* = 4). **(C)** Blood vessels were visible until day 10. Arrows indicate blood vessels in liver organoids. H&E staining of liver organoids (*n* = 4). Scale bars, 100 μm. **(D)** Immunofluorescence images of liver organoids at day 10 of culture showing retention of cell type specific markers HNF4A (hepatocytes), CD31 (endothelial cells), CD68 (kupffer cells) and α-SMA (stellate cells). Scale bar: 10 μm. **(E)** UMAP embedding of cell morphological features of human liver tissue. Percentage of hepatocytes, stellate cells, endothelial cells and kupffer cells were estimated by marker positivity. **(F)** ALT and AST levels in organoid culture supernatants treated with different concentrations of APAP (*n* = 4). **(G)** The levels of LDH in organoid culture supernatants treated with different treatments (*n* = 4). **(H)** Immunoblotting analysis of Bcl-2 and Bax levels in organoids under different treatment conditions (*n* = 4). *p < 0.05, ***p < 0.001, ****p < 0.0001

To validate these findings in a human-relevant system, we established human liver organoids from healthy donor tissue. These organoids recapitulated key features of human liver cellular composition and architecture. Using this platform, we developed an APAP-induced ALI model and a monocyte chemotaxis model (Fig. 7A). Organoids were cultured under conditions that preserve the native multicellular architecture. Within 7–10 days, organoids formed three-dimensional structures (Fig. 7B). Histological analysis by H&E staining revealed organized epithelial-like layers and internal cavities, recapitulating key structural features of the native liver (Fig. 7C). Immunofluorescence characterization confirmed the retention of multiple hepatic cell types. The organoids contained abundant HNF4α^+^ hepatocytes, CD31^+^ endothelial cells, α-SMA^+^ hepatic stellate cells and CD68^+^ macrophages, demonstrating the retention of both stromal and resident immune compartments (Fig. 7D). These cell ratios match previously determined cell distribution through scRNA-seq (Fig. 7E). This organoid model provides a humanized, multicellular platform that retains the critical cellular components and architectural cues necessary for modeling APAP-induced hepatocyte injury and monocyte chemotaxis.

Furthermore, APAP treatment of organoids led to paradoxical decreases in supernatant ALT/AST activity (Fig. 7F), reflecting direct inactivation of enzyme active sites by the toxic metabolite N-acetyl-p-benzoquinone imine (NAPQI)—a phenomenon consistent with in vitro hepatotoxicity models. Concurrently, LDH release increased (Fig. 7G), anti-apoptotic Bcl2 decreased, and pro-apoptotic Bax increased (Fig. 7H), confirming successful establishment of APAP-induced injury.

### VSIG4^+^ Mφ mitigate APAP-induced injury in human liver organoids via NF-κB-CCL2 suppression

**Fig. 8:**
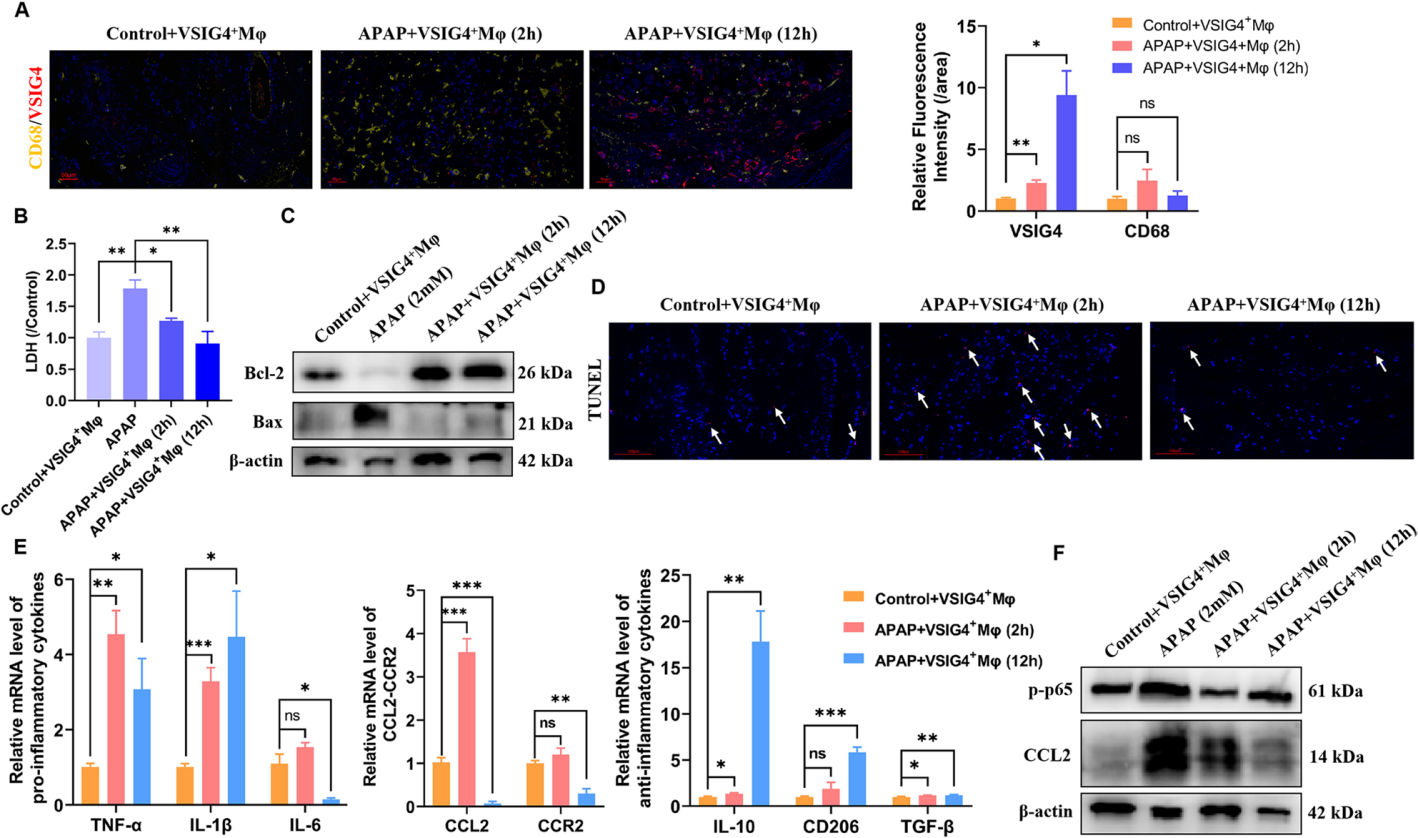
Human liver organoid ALI model confirms VSIG4^+^ MoMFs-mediated protection via NF-κB-CCL2 suppression. **(A)** Immunofluorescence staining and statistical results for markers of various cell types (CD68 and VSIG4) in liver organoids with different treatments. Scale bar: 100 μm. **(B)** The levels of LDH in organoid culture supernatants treated with different treatments (*n* = 4). **(C)** Immunoblotting analysis of Bcl-2 and Bax levels in organoids under different treatment conditions (*n* = 4). **(D)** Representative TUNEL staining images of organoids in each group (*n* = 4). Scale bar: 100 μm. **(E)** The relative mRNA expression levels of genes related to inflammation (IL1β, IL-6 and TNF-α), chemotaxis (CCL2 and CCR2) and anti-inflammation (IL10, CD206 and TGF-β) (*n* = 4). **(F)** Immunoblotting analysis of p-P65 and CCL2 levels in organoids under different treatment conditions (*n* = 3). *p < 0.05, **p < 0.01, ***p < 0.001, ns: no significant difference

Furthermore, using the ALI model based on liver organoids, we co-cultured it with THP-1 cells to establish a monocyte chemotaxis model. Addition of VSIG4^+^ Mφ significantly increased intra-organoid VSIG4^+^ cells (Fig. 8A), reduced LDH release (Fig. 8B), restored Bcl2/Bax balance (Fig. 8C), and decreased overall apoptosis (Fig. 8D). Mechanistically, VSIG4^+^ Mφ co-culture reduced pro-inflammatory cytokine expression, suppressed CCL2-CCR2 axis components, and elevated anti-inflammatory mediators (Fig. 8E). Protein-level analysis confirmed reduced CCL2 expression and p65 phosphorylation (Fig. 8F). These human organoid findings not only validate the therapeutic efficacy of VSIG4^+^ Mφ in a human-relevant system but also confirm that VSIG4-mediated suppression of CCL2-CCR2 axis represents a conserved mechanistic pathway across species.

## Discussion

The rapid progression from ALI to ALF remains one of the most formidable challenges in hepatology, with limited therapeutic options beyond transplantation [1, 21]. Macrophage-based therapies have emerged as promising candidates for intercepting this trajectory [22], yet their clinical translation has been hindered by subset imprecision and insufficient humanized validation. Through the integration of single-cell transcriptomics, reversible immunomagnetic isolation, and cross-species disease modeling, this study establishes VSIG4^+^ MoMFs as a therapeutically tractable cell subset for ALI and defines NF-κB-CCL2 axis suppression as a conserved mechanism.

These findings suggest that, despite partial infiltration of VSIG4^+^ MoMFs, the progression of ALI is characterized by the depletion of VSIG4^+^ KCs and the infiltration of a large number of inflammatory cells, which are predominantly VSIG4-negative. This depletion coincides with the establishment of an overactivated inflammatory microenvironment — consistent with previous observations that VSIG4^+^ Mφ loss represents a key immunological event in ALI-to-ALF transition [23]. VSIG4 functions as a complement receptor and immune checkpoint molecule, suppressing T-cell responses, inducing regulatory macrophage polarization, and maintaining hepatic immune tolerance [10, 11]. Notably, the increase in IL-10 levels observed after VSIG4^+^ Mφ transfer may further reinforce this immunoregulatory influence on adaptive immunity, as IL-10 potently inhibits effector T-cell responses and promotes regulatory T-cell expansion. Although adaptive immune parameters were not directly assessed in this study, this potential link warrants future investigation. VSIG4^+^ Mφ contribute to pathogen clearance, protection against alcoholic liver disease, and immunosuppressive microenvironment regulation [24–26]. However, their therapeutic application in ALI has not been systematically explored prior to this study.

To enable such therapy, we first developed a non-destructive isolation platform. At the cell procurement level, conventional immunomagnetic technologies enable capture but not release of target cells, limiting therapeutic utility [27]. Our MNP@Oligo-VSIG4 system, incorporating an Endo V-cleavable oligonucleotide linker, achieves rapid isolation (> 80% capture efficiency) with high viability (> 95%) and subsequent non-destructive bead detachment. The 15-minute capture and release protocol compares favorably with flow cytometric sorting (which requires larger sample volumes and can compromise viability) and represents a significant advance toward GMP-compliant cell manufacturing [28]. This platform is readily adaptable to other immune cell subsets, offering broad applicability for cell therapy development.

In terms of molecular mechanisms, we demonstrate that VSIG4^+^ Mφ suppress NF-κB p65 phosphorylation, downregulate CCL2 expression, and diminish CCR2^+^ monocyte/macrophage recruitment. The CCL2-CCR2 axis is well-established as a critical driver of inflammatory amplification in ALI [16, 17, 29]. Our transcriptomic and protein-level analyses confirm that VSIG4^+^ Mφ interrupt this pathway, aligning with reports that VSIG4 inhibits NF-κB signaling in metabolic liver disease and myocardial ischemia [18, 19]. Studies have shown that, as a transmembrane complement receptor, VSIG4 recruits the tyrosine phosphatase SHP-1 to the Toll-like receptor (TLR) signaling complex, thereby dephosphorylating key signaling intermediates (such as TRAF6 and IRAK1) and inhibiting the downstream phosphorylation cascade that leads to NF-κB activation [10]. Our data indicate reduced p65 phosphorylation levels and downregulated expression of NF-κB target genes, which are fully consistent with this established signaling pathway. Importantly, the protective effect of VSIG4^+^ Mφ was not solely attributable to transient phagocytic clearance of inflammatory mediators; rather, by interrupting the CCL2-CCR2 amplification loop, these cells attenuated the feed-forward recruitment of pro-inflammatory monocytes, thereby exerting a more sustained regulatory influence on the inflammatory cascade. Traditional AAMs are typically generated through in vitro polarization induced by cytokines (IL-4/IL-13), but these “M2a” macrophages exhibit phenotypic plasticity and may revert to a pro-inflammatory state when encountering the complex, dynamic environment of a damaged liver [30]. In contrast, VSIG4^+^ Mφ represent a naturally occurring and epigenetically fixed immunoregulatory subset that may exhibit greater functional stability in vivo and yield more predictable therapeutic outcomes. Our in vitro LPS challenge experiments support this notion, demonstrating that VSIG4^+^ Mφ are refractory to classical pro-inflammatory polarization. However, definitive evidence of long-term phenotypic stability in vivo would require lineage-tracing or long-term studies in chronic liver injury models, which represent an important direction for future investigation. Furthermore, future head-to-head comparisons between VSIG4^+^ macrophages and in vitro-polarized M2 macrophages, under both acute and chronic injury conditions, will be necessary to determine whether the inherent stability of VSIG4^+^ macrophages translates into superior safety and efficacy profiles.

To validate the translational relevance of these mechanisms in a human context, we established a human liver organoid model. Three-dimensional (3D) liver organoids have emerged as promising tools that recapitulate key structural and functional aspects of the native liver, providing a superior platform for disease modeling and therapeutic evaluation [31]. Primary human hepatocytes are scarce and rapidly dedifferentiate, while interspecies differences confound translational predictions [32]. Recent advances in organoid technology have enabled more physiologically relevant disease modeling [33], but liver organoids incorporating immune components remain challenging [34]. Our organoid system, derived from healthy human liver tissue, retains cellular heterogeneity and enables modeling of both APAP-induced hepatocyte injury and monocyte chemotaxis. In conventional organoid co-cultures, added immune cells are often confined to the periphery; however, our organoids preserve native vascular and sinusoidal architecture, which facilitates the infiltration of monocyte/macrophage cells into deeper parenchymal zones. Accordingly, we detected CCR2^+^ cells within the interior of APAP-treated organoids after co-culture, confirming that the THP-1 cells were not restricted to the surface (Supplementary Fig. 8). While this structural feature mitigates the penetration limitation, we acknowledge that the short exposure time and the absence of a fully competent immune system are constraints of this model. The convergent findings between murine models and human organoids provide robust evidence that VSIG4^+^ Mφ-mediated NF-κB-CCL2 suppression represents a conserved mechanism, substantially strengthening translational rationale. It is worth noting that while mouse models can demonstrate in vivo efficacy, they cannot prove that the therapeutic mechanism acts directly on human cells. Our organoid system differs from previous studies [35] on liver organoids in that it integrates human liver tissue (preserving part of the liver’s morphology and immune microenvironment) with the human monocyte THP-1 cell line, thereby overcoming this limitation. However, THP-1 cells are a transformed cell line and may not fully replicate primary macrophage biology. Future validation using primary human liver macrophages or blood-derived monocytes will therefore be essential to confirm these findings in a more physiologically authentic setting. In this human system, we observed that VSIG4^+^ Mφ can inhibit the NF-κB-CCL2 signaling pathway. This finding confirms that the key molecular mechanism is conserved in humans, thereby significantly reducing the risk associated with clinical translation.

Despite these advances, several limitations warrant consideration. First, while MNP@Oligo-VSIG4 isolation efficiency has been validated in cell lines and bone marrow-derived cells, performance in complex clinical samples (e.g., peripheral blood, hepatic perfusate) requires further optimization. Second, the in vivo distribution, persistence, and interactions of adoptively transferred VSIG4^+^ Mφ with other hepatic immune cells (neutrophils, NK cells, dendritic cells) remain incompletely characterized. Third, our therapeutic efficacy data are restricted to the acute phase of liver injury. Because the infused cells are terminally differentiated primary BMDM, they are expected to survive only transiently in vivo and to exert protection via acute paracrine mechanisms. Accordingly, dedicated long-term tracking studies using stably labeled cells and chronic injury models will be required to fully assess the persistence, distribution, and eventual clearance of these cells.

## Conclusions

This study establishes VSIG4^+^ Mφ as a precisely defined, therapeutically efficacious macrophage subcluster for ALI. Compared with unselected BMDM, which failed to confer protection and even exacerbated pro-inflammatory signatures, VSIG4^+^ Mφ consistently mitigated liver injury by suppressing NF-κB-driven CCL2 expression, thereby interrupting the CCL2-CCR2 inflammatory amplification loop and reducing secondary monocyte/macrophage recruitment. The convergent findings in murine models and human liver organoids provide robust preclinical rationale for advancing VSIG4^+^ Mφ therapy toward clinical evaluation in ALI and potentially other inflammatory liver diseases.

## Supplementary Information

Below is the link to the electronic supplementary material.

Supplementary Material 1

Supplementary Material 2

## Data Availability

The datasets used and/or analysed during the current study are available from the corresponding author on reasonable request. Public datasets analyzed are available from GEO (GSE192742, GSE280515 and GSE280514).

## Abbreviations

AAMs: Alternatively activated macrophages
ALF: Acute liver failure
ALI: Acute liver injury
ALT: Alanine aminotransferase
APAP: Acetaminophen
AST: Aspartate aminotransferase
BMDM: Bone marrow-derived monocytes
CCL2: C-C motif chemokine ligand 2
CCR2: C-C motif chemokine receptor 2
DEGs: Differentially expressed genes
dI: Deoxyinosine
EDC: 1-ethyl-3-(3-dimethylaminopropyl) carbodiimide
Endo V: Endonuclease V
GEO: Gene Expression Omnibus
HNF4α: Hepatocyte nuclear factor 4 alpha
IL: Interleukin
KCs: Kupffer cells
LAMs: Lipid-associated macrophages
LDH: Lactate dehydrogenase
LPS: Lipopolysaccharide
M-CSF: Macrophage colony-stimulating factor
MCP-1: Monocyte chemoattractant protein-1
MES: 2-(N-morpholino) ethanesulfonic acid
MNP: Magnetic nanoparticle
MoMFs: Monocyte-derived macrophages
NAPQI: N-acetyl-p-benzoquinone imine
NF-κB: Nuclear factor kappa B
NHS: N-hydroxysuccinimide
scRNA-seq: Single-cell RNA sequencing
α-SMA: Alpha smooth muscle actin
TGF-β: Transforming growth factor-beta
TNF: Tumor necrosis factor
VSIG4: V-set and immunoglobulin domain containing 4
WGCNA: Weighted gene co-expression network analysis

## References

[1] Lemmer P, Sowa JP, Bulut Y, Strnad P, Canbay A. Mechanisms and aetiology-dependent treatment of acute liver failure. Liver Int. 2025;45:e15739. 37752801 10.1111/liv.15739 PMC11815625

[2] Sitbon A, Delmotte PR, Pistorio V, Halter S, Gallet J, Gautheron J, Monsel A. Mesenchymal stromal cell-derived extracellular vesicles therapy openings new translational challenges in immunomodulating acute liver inflammation. J Transl Med. 2024;22:480. 38773651 10.1186/s12967-024-05282-9 PMC11106935

[3] Shang AQ, Yan H, Xiang Z, Chen JQ, Jiang B, Jiang C, Ling B, Wu J, Chinese Consortium for the Study of Hepatitis E. Serum exosome-derived ALDH1A1 can greatly predict the prognosis of patients with hepatitis E virus-related acute liver failure. Hepatobiliary Pancreat Dis Int. 2025;24:170–6. 39753426 10.1016/j.hbpd.2024.12.007

[4] Artru F, Trovato F, Morrison M, Bernal W, McPhail M. Liver transplantation for acute-on-chronic liver failure. Lancet Gastroenterol Hepatol. 2024;9:564–76. 38309288 10.1016/S2468-1253(23)00363-1

[5] Jothimani D, Marannan NK, Rela M. Acute liver failure and liver transplantation. Indian J Gastroenterol. 2025;44:298–310. 39964603 10.1007/s12664-024-01708-w

[6] He K, Zhu XJ, Shi YP, Huang WJ, Yang TH, Xi ZF, Li QG, Sun HY, Qian LJ, Chen XS, et al. Treatment of liver cirrhosis using hepatocyte-derived liver progenitor-like cells: a prospective, open-label, single-arm, safety trial. Cell Discov. 2025;11:88. 41193418 10.1038/s41421-025-00831-y PMC12589444

[7] Starkey Lewis P, Campana L, Aleksieva N, Cartwright JA, Mackinnon A, O’Duibhir E, Kendall T, Vermeren M, Thomson A, Gadd V, et al. Alternatively activated macrophages promote resolution of necrosis following acute liver injury. J Hepatol. 2020;73:349–60. 32169610 10.1016/j.jhep.2020.02.031 PMC7378576

[8] Xiao P, Han X, Huang Y, Yang J, Chen L, Cai Z, Hu N, Cui W, Huang W. Reprogramming macrophages via immune cell mobilized hydrogel microspheres for osteoarthritis treatments. Bioact Mater. 2024;32:242–59. 37869722 10.1016/j.bioactmat.2023.09.010 PMC10589729

[9] Candela ME, Addison M, Aird R, Man TY, Cartwright JA, Ashmore-Harris C, Kilpatrick AM, Starkey Lewis PJ, Drape A, Barnett M, et al. Cryopreserved human alternatively activated macrophages promote resolution of acetaminophen-induced liver injury in mouse. NPJ Regen Med. 2025;10:5. 39843512 10.1038/s41536-025-00393-3 PMC11754469

[10] Li J, Diao B, Guo S, Huang X, Yang C, Feng Z, Yan W, Ning Q, Zheng L, Chen Y, Wu Y. VSIG4 inhibits proinflammatory macrophage activation by reprogramming mitochondrial pyruvate metabolism. Nat Commun. 2017;8:1322. 29109438 10.1038/s41467-017-01327-4 PMC5673889

[11] Liu B, Cheng L, Gao H, Zhang J, Dong Y, Gao W, Yuan S, Gong T, Huang W. The biology of VSIG4: Implications for the treatment of immune-mediated inflammatory diseases and cancer. Cancer Lett. 2023;553:215996. 36343787 10.1016/j.canlet.2022.215996

[12] Zheng S, Li S, Wang Q, Zao X, Li X, Qi W, Cheng F, Geng Y, Zhang P, Shi X, Ye Y. New perspectives in the treatment of liver cirrhosis: targeting macrophage regulatory mechanisms. J Transl Med. 2025;23:1201. 41174741 10.1186/s12967-025-07239-y PMC12577345

[13] Liu J, Zhang W, Chen L, Wang X, Mao X, Wu Z, Shi H, Qi H, Chen L, Huang Y, et al. VSIG4 Promotes Tumour-Associated Macrophage M2 Polarization and Immune Escape in Colorectal Cancer via Fatty Acid Oxidation Pathway. Clin Transl Med. 2025;15:e70340. 40405491 10.1002/ctm2.70340 PMC12098961

[14] Cho HY, Yang YG, Jeon Y, Lee CK, Choi I, Lee SW. VSIG4(+) peritoneal macrophages induce apoptosis of double-positive thymocyte via the secretion of TNF-alpha in a CLP-induced sepsis model resulting in thymic atrophy. Cell Death Dis. 2021;12:526. 34023853 10.1038/s41419-021-03806-5 PMC8139869

[15] Lu Q, Zhang Z, Ding X. Isolation Techniques of Micro/Nano-Scaled Species for Biomedical Applications. Adv Sci (Weinh). 2025;12:e2414109. 40411414 10.1002/advs.202414109 PMC12244519

[16] Mossanen JC, Krenkel O, Ergen C, Govaere O, Liepelt A, Puengel T, Heymann F, Kalthoff S, Lefebvre E, Eulberg D, et al. Chemokine (C-C motif) receptor 2-positive monocytes aggravate the early phase of acetaminophen-induced acute liver injury. Hepatology. 2016;64:1667–82. 27302828 10.1002/hep.28682

[17] Pozzi S, Satchi-Fainaro R. The role of CCL2/CCR2 axis in cancer and inflammation: The next frontier in nanomedicine. Adv Drug Deliv Rev. 2024;209:115318. 38643840 10.1016/j.addr.2024.115318

[18] Li Y, Sun JP, Wang J, Lu WH, Xie LY, Lv J, Li HX, Yang SF. Expression of Vsig4 attenuates macrophage-mediated hepatic inflammation and fibrosis in high fat diet (HFD)-induced mice. Biochem Biophys Res Commun. 2019;516:858–65. 31266632 10.1016/j.bbrc.2019.06.045

[19] Wang Y, Ding J, Song H, Teng Y, Fang X. VSIG4 regulates macrophages polarization and alleviates inflammation through activating PI3K/AKT and inhibiting TLR4/NF-kappaB pathway in myocardial ischemia-reperfusion injury rats. Physiol Int. 2022;109:356–70. 36057104 10.1556/2060.2022.00055

[20] Lu J, Gu X, Xue C, Shi Q, Jia J, Cheng J, Zeng Y, Chu Q, Yuan X, Bao Z, Li L. Glycyrrhizic acid alleviates concanavalin A-induced acute liver injury by regulating monocyte-derived macrophages. Phytomedicine. 2024;133:155586. 39159503 10.1016/j.phymed.2024.155586

[21] Wu PP, Shen XJ, Zheng SS. Cisplatin induces acute liver injury by triggering caspase-3/GSDME-mediated cell pyroptosis. Hepatobiliary Pancreat Dis Int. 2025;24:177–87. 39419722 10.1016/j.hbpd.2024.09.010

[22] Maiwall R, Kulkarni AV, Arab JP, Piano S. Acute liver failure. Lancet. 2024;404:789–802. 39098320 10.1016/S0140-6736(24)00693-7

[23] Duan Y, Chu H, Brandl K, Jiang L, Zeng S, Meshgin N, Papachristoforou E, Argemi J, Mendes BG, Wang Y, et al. CRIg on liver macrophages clears pathobionts and protects against alcoholic liver disease. Nat Commun. 2021;12:7172. 34887405 10.1038/s41467-021-27385-3 PMC8660815

[24] Reissing J, Lutz P, Frissen M, Ibidapo-Obe O, Reuken PA, Wirtz TH, Stengel S, Quickert S, Rooney M, Grosse K, et al. Immunomodulatory receptor VSIG4 is released during spontaneous bacterial peritonitis and predicts short-term mortality. JHEP Rep. 2022;4:100391. 34917912 10.1016/j.jhepr.2021.100391 PMC8666561

[25] Saris J, Li Yim AYF, Bootsma S, Lenos KJ, Franco Fernandez R, Khan HN, Verhoeff J, Poel D, Mrzlikar NM, Xiong L, et al. Peritoneal resident macrophages constitute an immunosuppressive environment in peritoneal metastasized colorectal cancer. Nat Commun. 2025;16:3669. 40246872 10.1038/s41467-025-58999-6 PMC12006467

[26] James SE, Chen S, Ng BD, Fischman JS, Jahn L, Boardman AP, Rajagopalan A, Elias HK, Massa A, Manuele D, et al. Leucine zipper-based immunomagnetic purification of CAR T cells displaying multiple receptors. Nat Biomed Eng. 2024;8:1592–614. 39715901 10.1038/s41551-024-01287-3 PMC11917073

[27] Vijayan Nair S. Isolation and release of circulating cells using cleavable oligonucleotide linkers in microfluidic devices: application for minimum residual disease (MRD) assessment in acute myeloid leukemia. [master’s thesis]. Raleigh (NC): North Carolina State University; 2014.

[28] Luce E, Messina A, Duclos-Vallee JC. Hepatic organoids as a platform for liver disease modeling and the development of novel therapies. Clin Res Hepatol Gastroenterol. 2025;49:102647. 40615111 10.1016/j.clinre.2025.102647

[29] Li T, Zeng H, Xian W, Cai H, Zhang J, Zhou S, Yang Y, Luo M, Zhu P. Maresin1 alleviates liver ischemia/reperfusion injury by reducing liver macrophage pyroptosis. J Transl Med. 2023;21:472. 37455316 10.1186/s12967-023-04327-9 PMC10351145

[30] Behan-Bush RM, Schrodt MV, Kilburg E, Liszewski JN, Bitterlich LM, English K, Klingelhutz AJ, Ankrum JA. Polychlorinated biphenyls induce immunometabolic switch of antiinflammatory macrophages toward an inflammatory phenotype. PNAS Nexus. 2025;4:pgaf100. 40191133 10.1093/pnasnexus/pgaf100 PMC11969150

[31] Zhao J, Zhi Y, Ren H, Wang J, Zhao Y. Emerging biotechnologies for engineering liver organoids. Bioact Mater. 2025;45:1–18. 39588483 10.1016/j.bioactmat.2024.11.002 PMC11585797

[32] Verstegen MMA, Coppes RP, Beghin A, De Coppi P, Gerli MFM, de Graeff N, Pan Q, Saito Y, Shi S, Zadpoor AA, van der Laan LJW. Clinical applications of human organoids. Nat Med. 2025;31:409–21. 39901045 10.1038/s41591-024-03489-3

[33] Sljukic A, Green Jenkinson J, Niksic A, Prior N, Huch M. Advances in liver and pancreas organoids: how far we have come and where we go next. Nat Rev Gastroenterol Hepatol. 2026;23:44–64. 41073688 10.1038/s41575-025-01116-1

[34] Cao Q, Cai C, Wang C, Li L, Liu J, Zhang J, Rong M, Ren J, Han Y, Zhang J, Han X. Zengmian Yiliu formula suppresses cell cycle in immune-rich ovarian cancer patient-derived organoids. Phytomedicine. 2025;141:156721. 40215819 10.1016/j.phymed.2025.156721

[35] Ye Z, Yan J, Wang Y, Lin Y, Wang C, Yang Q, Ji T, Zhou E, Zheng Q, Zhong D, et al. Three-dimensional bioprinted hiHeps hepatorganoids with enhanced hepatic functions for the treatment of liver failure and promotion of liver regeneration. Bioact Mater. 2026;58:550–73. 41502981 10.1016/j.bioactmat.2025.12.024 PMC12771358

