# Supplementary material for: Adoptive transfer of VSIG4+ macrophages interrupts the CCL2-CCR2 inflammatory amplification loop to mitigate acetaminophen-induced acute liver injury in murine and human organoid models
Source: J Transl Med. 2026 Jul 20;24:933. doi: 10.1186/s12967-026-08631-y (PMC13386671; doi:10.1186/s12967-026-08631-y)
Supplement: Supplementary file 1 — Supplementary Material 1 [file 12967_2026_8631_MOESM1_ESM.docx]

**Additional file 1**

**Adoptive Transfer of VSIG4^+^ Macrophages Interrupts the CCL2-CCR2 Inflammatory Amplification Loop to Mitigate Acetaminophen-induced Acute Liver Injury in Murine and Human Organoid Models**

Biao Duan ^1^**^†^**, Jun Chen ^2^**^†^**, Guan Liu ^3^**^†^**, Jiacheng Lin ^4^, Shiyu Yang ^2^, Xiaoni Kong ^4, *^, Weifang Rong ^5, *^, Weifeng Tan ^1, *^

**This file includes:**

Additional Figures:

Fig. S1. Analysis of scRNA-seq data on distribution of VSIG4^+^ cells in human liver tissue cells clusters.

Fig. S2. Analysis of scRNA-seq data on distribution of VSIG4^+^ cells in mouse liver tissue cells clusters.

Fig. S3. Construction and Characterization of MNP@Oligo-VSIG4.

Fig. S4. Optimization of the isolation buffer and validation of the isolation of VSIG4^+^ Cells.

Fig. S5. Flow cytometry gating strategy.

Fig. S6. VSIG4^+^ Mφ suppress hepatic inflammation.

Fig. S7. VSIG4^+^ Mφ inhibit CCL2-mediated immune cells recruitment by suppressing the NF-κB pathway.

Fig. S8. Immunofluorescence detection of CCR2^+^ myeloid cells inside liver organoids after APAP treatment.

Additional Tables:

Table S1. Clinical characteristics of healthy donors and ALI patients.

Table S2. List of the first and secondary antibodies.

Table S3. List of the primer of RT-qPCR performed in the experiments.


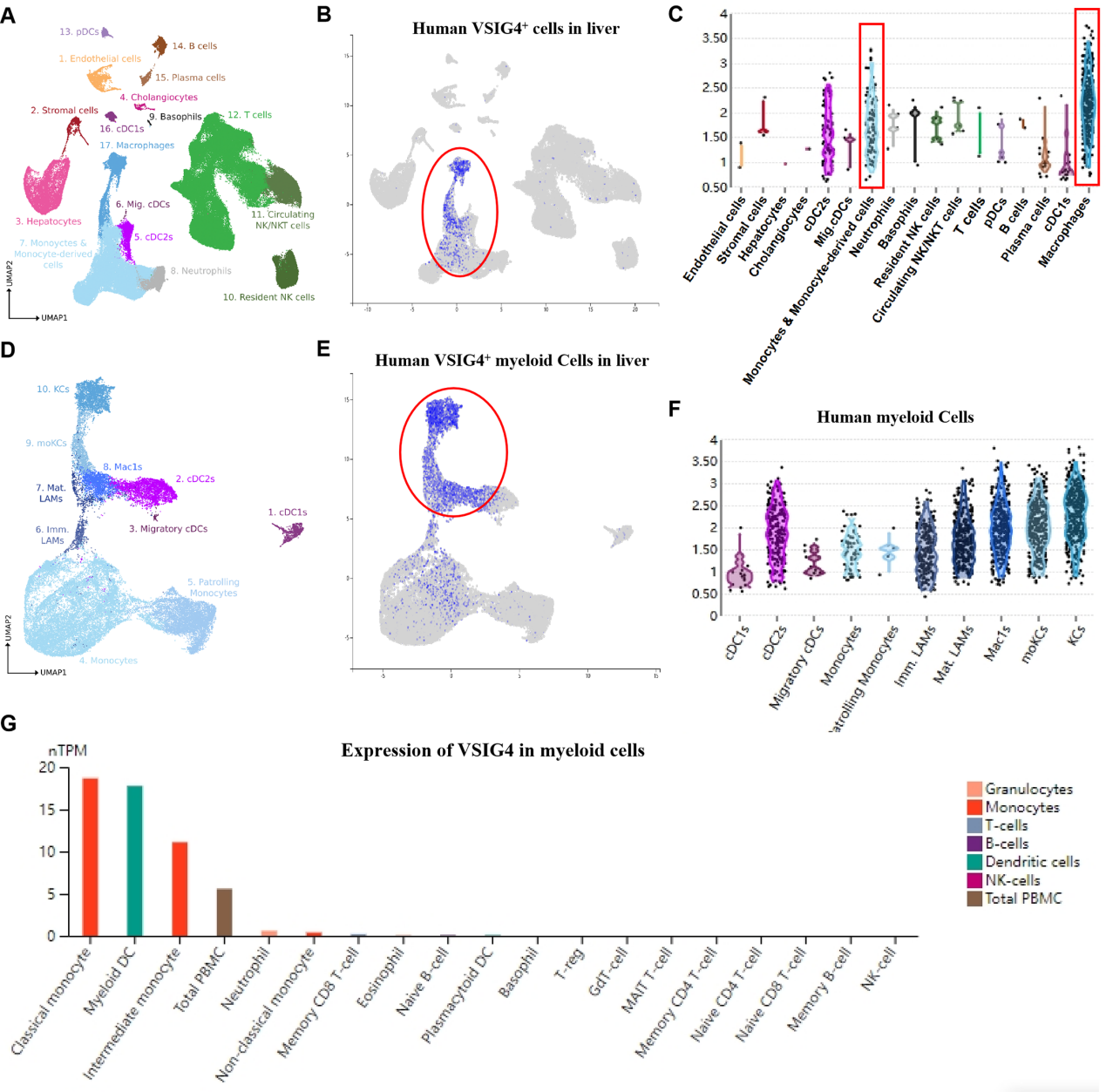


**Figure S1. Analysis of scRNA-seq data on distribution of VSIG4^+^ cells in human liver tissue cells clusters.** (A) The annotated UMAP highlighted seventeen distinct clusters formed in human liver tissue cells. (B) and (C) Display the UMAP plot and Violin plot of human liver tissue cells stratified by VSIG4 expression to highlight the distribution of VSIG4^+^ cells. (D) The annotated UMAP highlighted ten distinct clusters formed in myeloid cells of human liver tissue. (E) and (F) Display the UMAP plot and Violin plot of myeloid cells of human liver tissue stratified by VSIG4 expression to highlight the distribution of VSIG4^+^ cells. (G) The expression of VSIG4 in myeloid cells of human liver tissue from HPA datasets (https://www.proteinatlas.org/ENSG00000155659-VSIG4/single+cell).


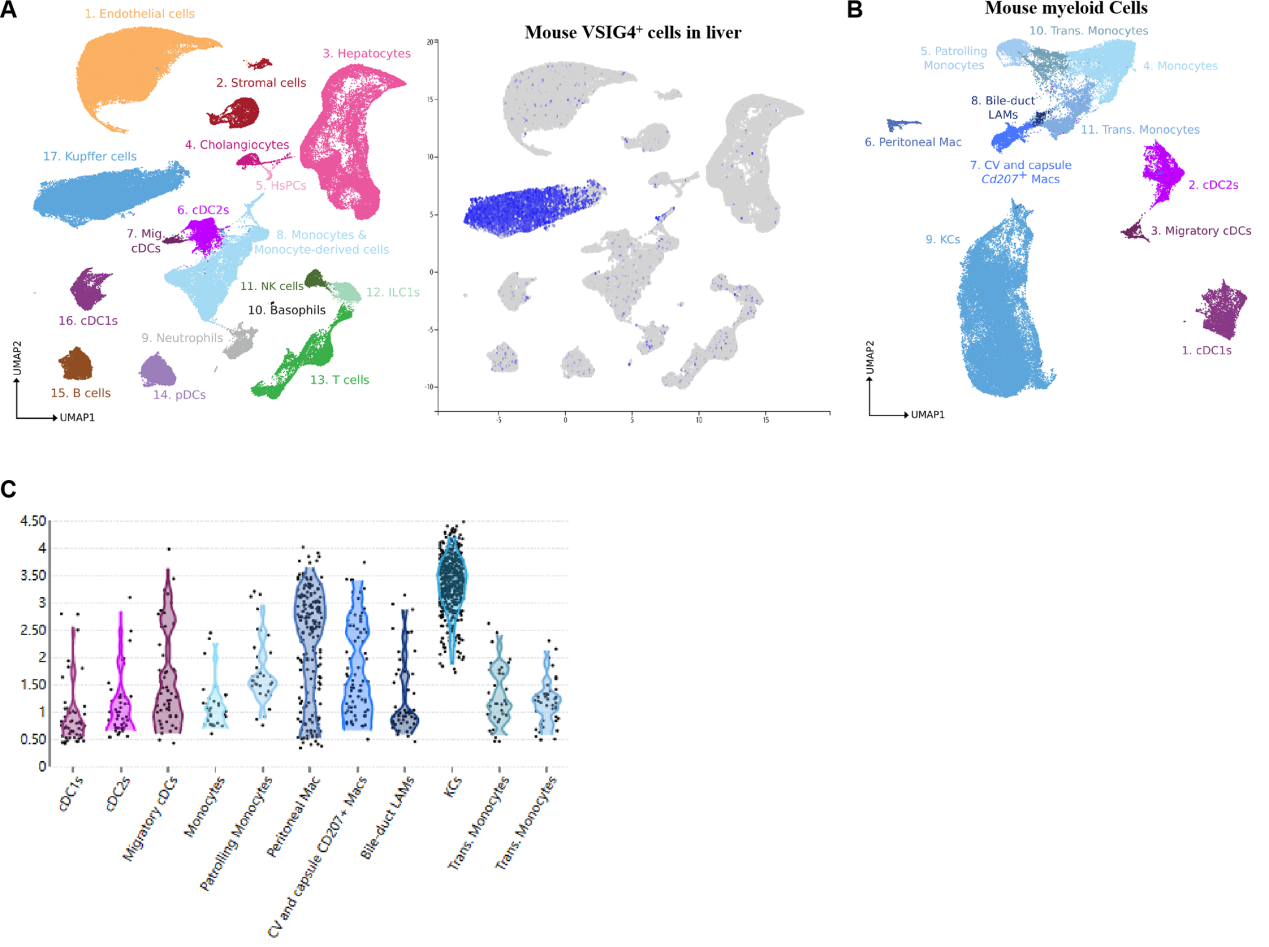


**Figure S2. Analysis of scRNA-seq data on distribution of VSIG4^+^ cells in mouse liver tissue cells clusters.** (A) The annotated UMAP highlighted seventeen distinct clusters formed in mouse liver tissue cells. Display the UMAP plot of mouse liver tissue cells stratified by VSIG4 expression to highlight the distribution of VSIG4+ cells. (B) The annotated UMAP highlighted eleven distinct clusters formed in myeloid cells of mouse liver tissue. (C) Display the UMAP plot of myeloid cells of mouse liver tissue stratified by VSIG4 expression to highlight the distribution of VSIG4+ cells.


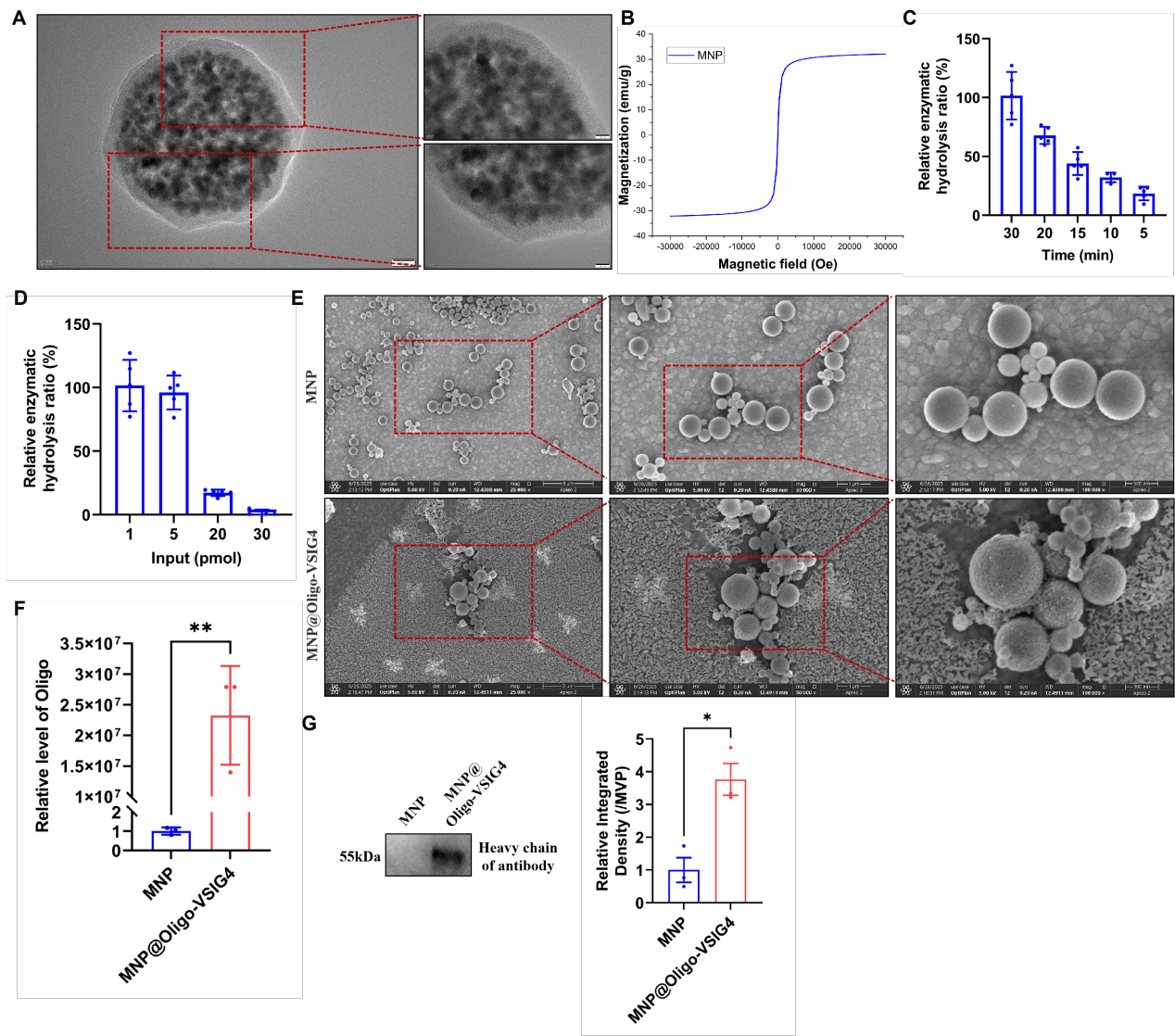


**Figure S3. Construction and Characterization of MNP@Oligo-VSIG4.** (A) Representative SEM images of MNPs at high magnification. Scale bar: 20 nm and 10 nm. (B) Magnetic hysteresis curves of MNPs. (C) and (D) Efficiency of Endo V hydrolysis of oligonucleotides (oligo) at different incubation times and reaction concentrations (n=5). (E) Representative SEM images of MNP and MNP@Oligo-VSIG4. Scale bar: 3 μm, 1 μm and 500 nm. (F) and (G) Comparison of oligo and antibody levels between MNP and MNP@Oligo-VSIG4 (n=3).*p < 0.05, **p < 0.01.


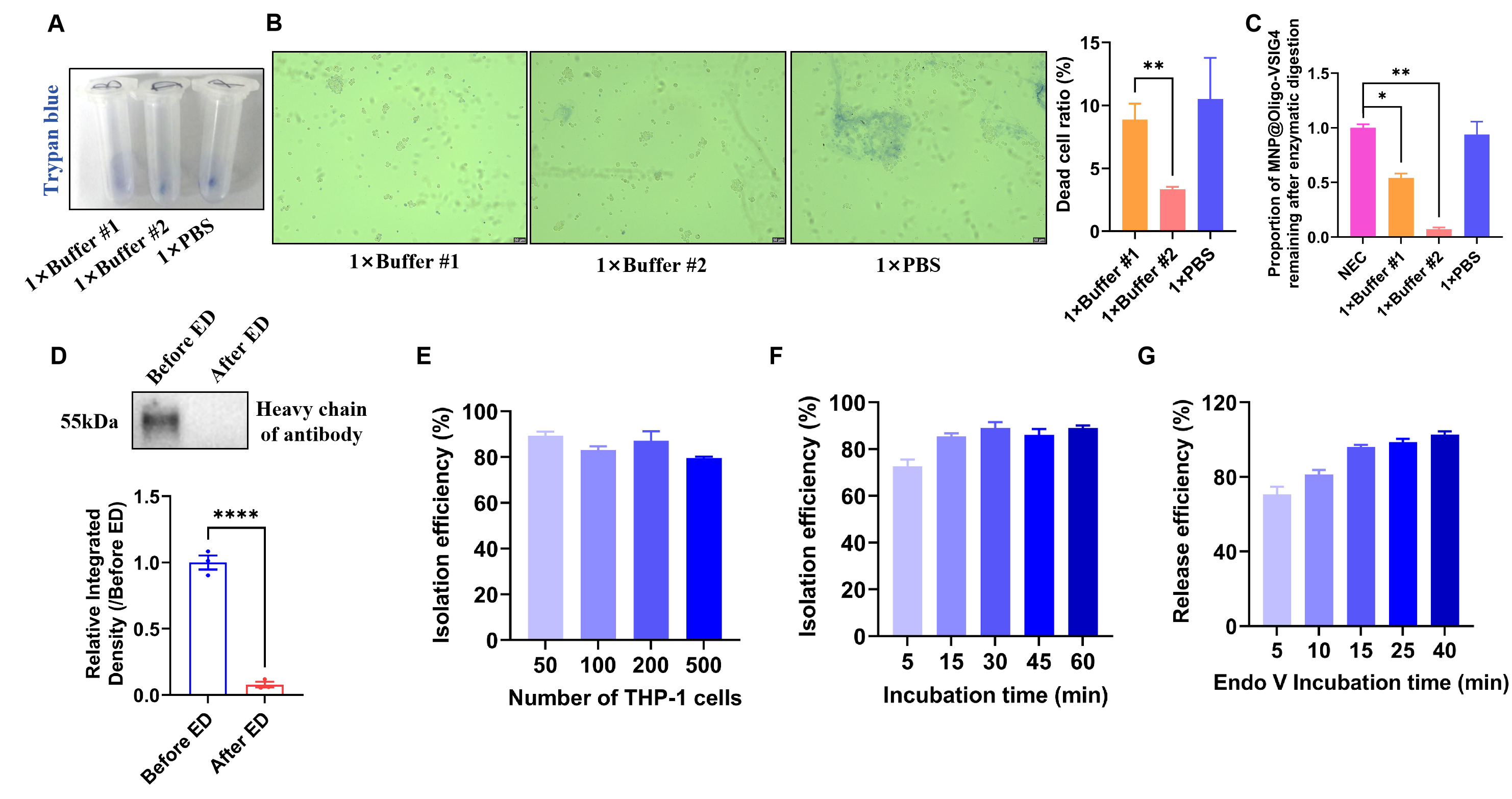


**Figure S4. Optimization of the isolation buffer and validation of the isolation of VSIG4^+^ Cells.** (A) and (B) Detection of viable cell levels and cell dispersion in each group using Tapanin blue staining (n=3). (C) The amount of intact MNP@Oligo-VSIG4 remaining after enzymatic digestion (ED) in each buffer (n=3). (D) Comparison of antibody levels of MNP@Oligo-VSIG4 between before and after ED (n=3). (E) and (F) Isolation efficiency of MNP@Oligo-VSIG4 for THP-1 cells under different cell concentrations and incubation times (n=3). (G) Release efficiency of THP-1 cells under different ED times (n=3). *p < 0.05, **p < 0.01, ****p < 0.0001.


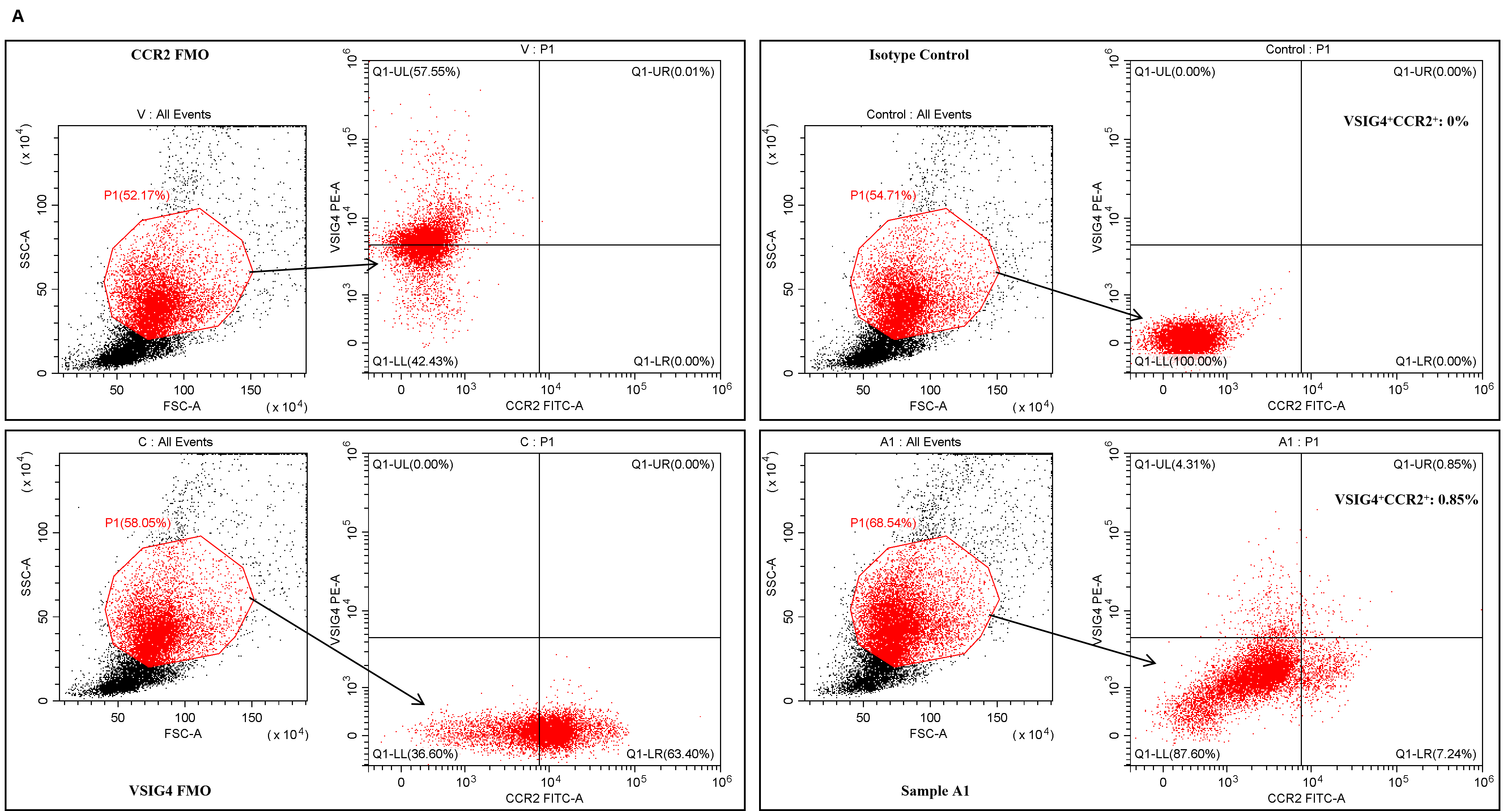


**Figure S5. Flow cytometry gating strategy.** (A) The gating strategy proceeded as follows: debris was excluded by forward scatter (FSC-A) versus side scatter (SSC-A). Singlet cells were then analyzed for VSIG4 and CCR2 expression. The VSIG4+ gate was set using a fluorescence-minus-one (FMO) control tube stained with CCR2-AF568 only (without VSIG4-AF488), and the CCR2^+^ gate was set using an FMO control tube stained with VSIG4-AF488 only (without CCR2-AF568). Isotype-matched control antibodies were used in parallel to confirm the specificity of both gates. The final dot plot displays VSIG4 versus CCR2, with VSIG4^+^，CCR2^+^ (recruited monocyte-derived) and VSIG4^+^CCR2^+^ (recruited monocyte-derived VSIG4^+^ cells) macrophage populations indicated. This same gating strategy was applied to all bone marrow samples. n=5 mice per group.


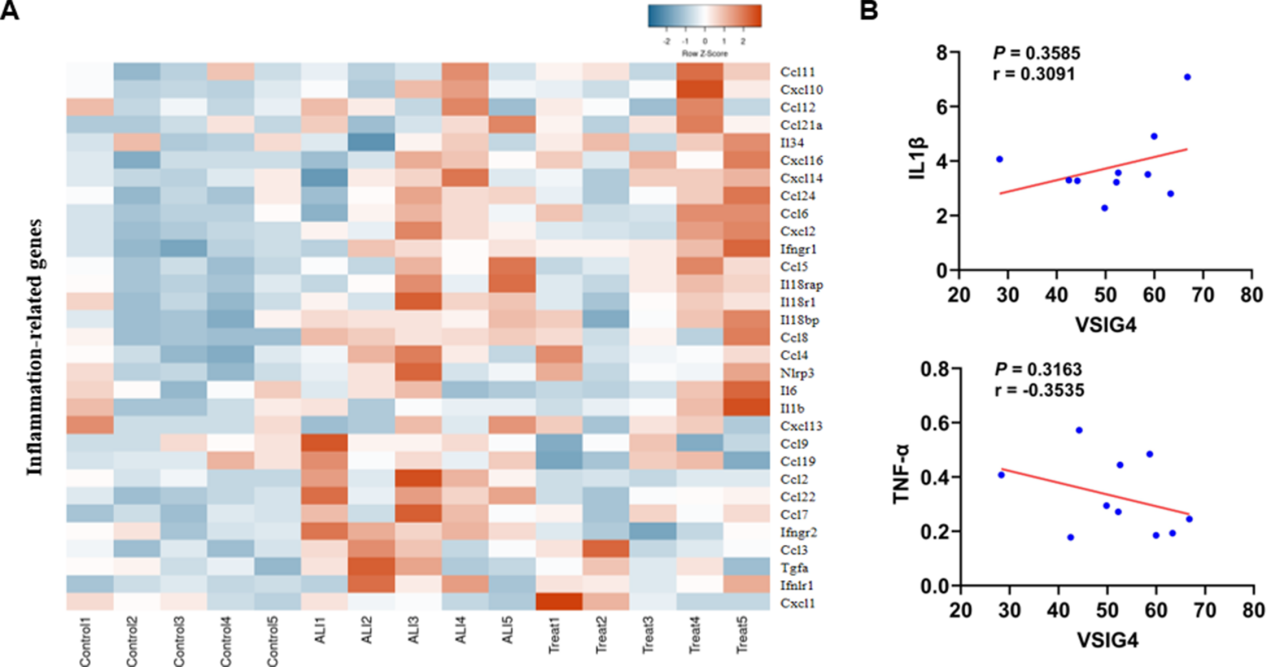


**Figure S6. VSIG4^+^ Mφ suppress hepatic inflammation.** (A) Heatmap show the intrahepatic expression of members of inflammation (n=5). (B) Analysis of the correlation between the mRNA expression of inflammatory factors (IL1β and TNF-α) and VSIG4 expression in the liver tissues of mice in each group (n=10).


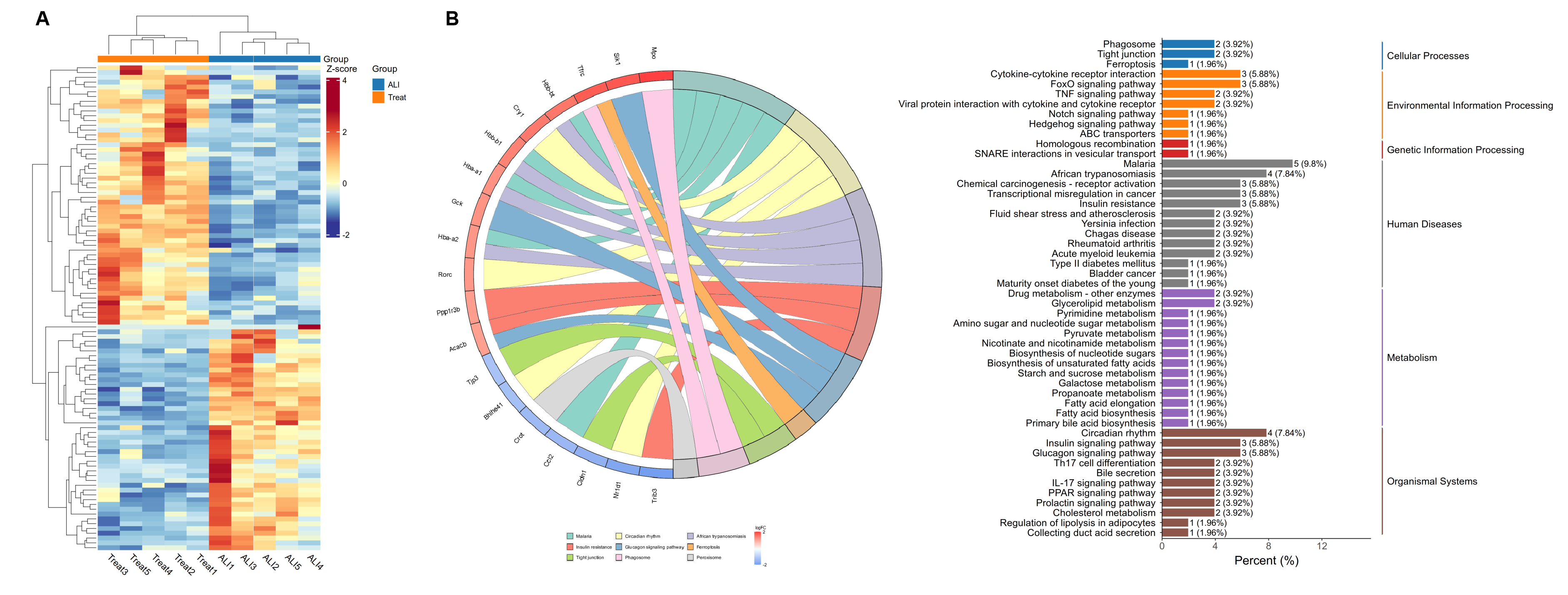


**Figure S7. VSIG4^+^ Mφ inhibit CCL2-mediated immune cells recruitment by suppressing the NF-κB pathway**. (A) Heatmap of DEGs in VSIG4^+^ Mφ treatment group compared with ALI group (n=5). (B) The KEGG enrichment chordplot and barplot enriched among the DEGs.


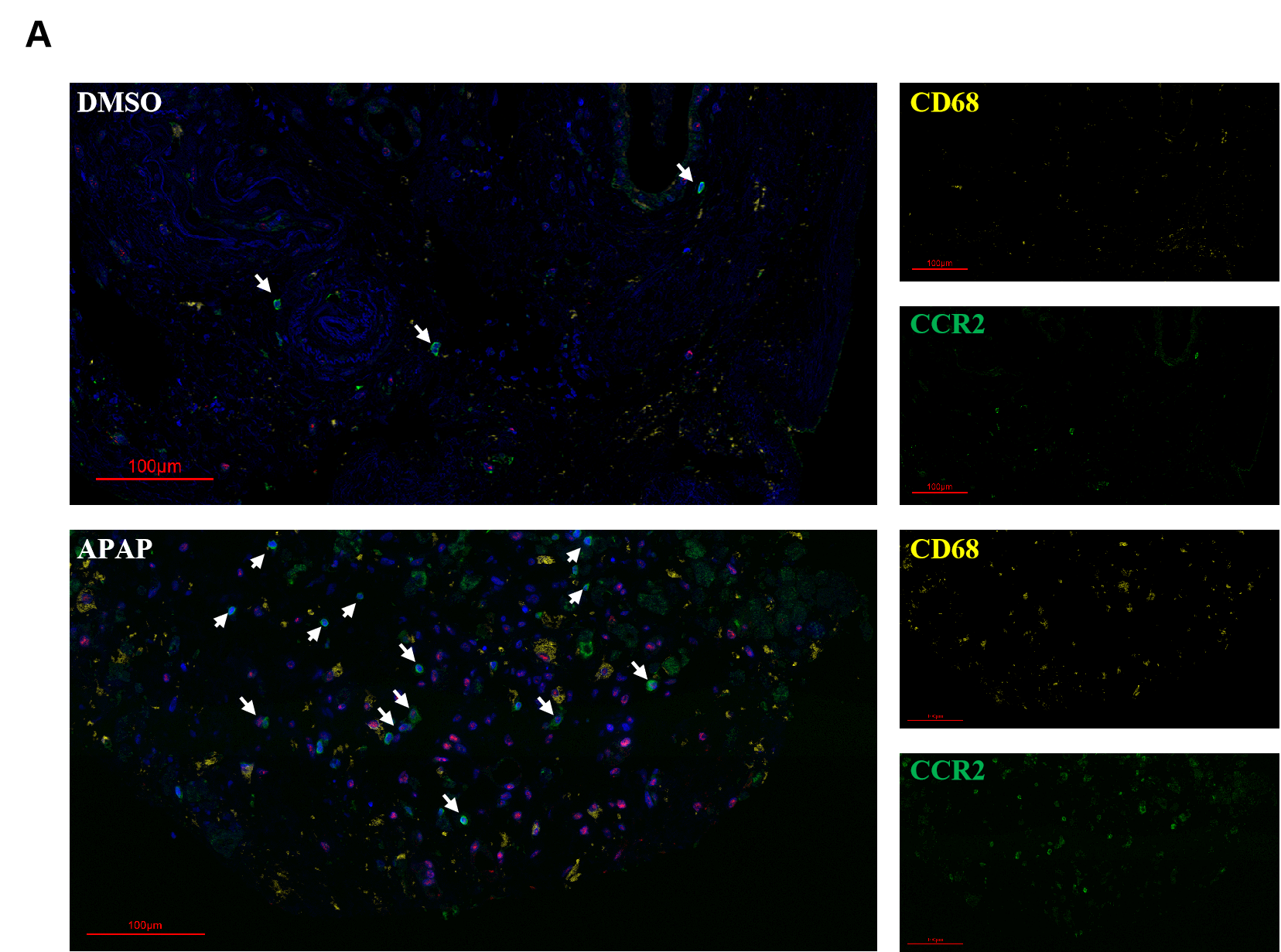


**Figure S8. Immunofluorescence detection of CCR2^+^ myeloid cells inside liver organoids after APAP treatment.** (A) Representative immunofluorescence images of liver organoids from the DMSO (control) and APAP-treated groups, stained for CCR2 (green) and CD68 (yellow). Nuclei were counterstained with DAPI (blue). White arrows indicate CCR2^+^ cells located within the organoid parenchyma, demonstrating that monocyte-derived macrophages can infiltrate into the interior of the organoids following APAP-induced injury. Scale bar, 100 μm.

**Table S1. Clinical characteristics of healthy donors and ALI patients.**

|  | Healthy donors | ALI patients |
| --- | --- | --- |
| Gender (M/F) | 2/1 | 1/2 |
| Age, year | 57.3 ± 15.4 | 56.0 ± 8.5 |
| BMI, kg/m^2^ | 23.6 ± 4.0 | 22.3 ± 1.1 |
| AST (U/L) | 13.3 ± 3.4 | 156.3 ± 189.3* |
| ALT (U/L) | 18.2 ± 3.4 | 142.6 ± 143.4* |
| White Blood Cell (10^9^/L） | 5.97±2.01 | 7.37±1.55 |
| Lymphocytes (%) | 26.73±9.00 | 39.13±9.52 |
| Neutrophils (%) | 62.67±7.28 | 51.30±6.06 |
| Monocytes (%)‌‌‌ | 6.70±1.21 | 7.47±4.18 |

| **Table S2. List of the first and secondary antibodies.** | | | | |
| --- | --- | --- | --- | --- |
| **Antibodies** | **Source** | **Host** | **Identifier** | **Dilution** |
| VSIG4 | Abmart | Mouse | M044899 | 1:1000 (IB) /1:200 (IF)/ 1:50 (FC) |
| VSIG4 | Abmart | Rabbit | PC0479 | 1:200 (IF) |
| CCR2 | Servicebio | Rabbit | GB11326 | 1:800 (IF) |
| F4/80 | Servicebio | Rabbit | GB113373 | 1:500 (IF)/1:500 (IHC) |
| Bcl2 | Abmart | Rabbit | T40056S | 1:1000 (IB) |
| Bax | Selleck | Rabbit | F0037 | 1:1000 (IB) |
| CD68 | Huilan biotech | Rabbit | ABB00011 | 1:500 (IF) |
| p-P65 | Abmart | Rabbit | [TB3675](https://www.ab-mart.com.cn/page.aspx?node= 77 &id= 329635" \t "https://www.ab-mart.com.cn/_blank) | 1:1000 (IB) |
| CCL2 | Abmart | Rabbit | [TD7577](https://www.ab-mart.com.cn/page.aspx?node= 77 &id= 23416" \t "https://www.ab-mart.com.cn/_blank) | 1:1000 (IB) |
| β-actin | Servicebio | Rabbit | GB15003 | 1:5000 (IB) |
| HNF4α | Proteintech | Rabbit | 26245-1-AP | 1:600 (IF) |
| CD31 | Abmart | Rabbit | [TA6191](https://www.ab-mart.com.cn/page.aspx?node= 77 &id= 21341" \t "https://www.ab-mart.com.cn/_blank) | 1:500 (IF) |
| α-SMA | Proteintech | Rabbit | 14395-1-AP | 1:600 (IF) |
| MPO | Servicebio | Rabbit | GB150006 | 1:500 (IHC) |
| HRP-conjugated Rabbit secondary antibody | Beyotime | Goat | A0208 | 1:5000 (IB) |
| HRP-conjugated mouse secondary antibody | Beyotime | Goat | A0216 | 1:5000 (IB) |
| Goat anti-Rabbit IgG(H+L) (Alexa Fluor® 568 Conjugate) | STARTER | Goat | S0B4007 | 1:500 (IF) |
| Goat anti-Mouse IgG(H+L) (Alexa Fluor® 488 Conjugate) | STARTER | Goat | S0B4010 | 1:500 (IF) |
| IB: Immunoblotting; IF: Immunofluorescence; FC: Flow Cytometry; IHC: Immunohistochemistry. | | | | |

| **Table S3. List of the primer of RT-qPCR performed in the experiments.** | | | |
| --- | --- | --- | --- |
| **Gene** | **Species** | **Forward (5'-3')** | **Reverse (5'-3')** |
| *IL-6* | *Homo* | CCTGAACCTTCCAAAGATGGC | TTCACCAGGCAAGTCTCCTCA |
|  | *Mus* | TAGTCCTTCCTACCCCAATTTCC | TTGGTCCTTAGCCACTCCTTC |
| *TNF-α* | *Homo* | GAGGCCAAGCCCTGGTATG | CGGGCCGATTGATCTCAGC |
|  | *Mus* | CCTGTAGCCCACGTCGTAG | GGGAGTAGACAAGGTACAACCC |
| *IL-1β* | *Homo* | TTCGACACATGGGATAACGAGG | TTTTTGCTGTGAGTCCCGGAG |
|  | *Mus* | GAAATGCCACCTTTTGACAGTG | TGGATGCTCTCATCAGGACAG |
| *IL-10* | *Homo* | GACTTTAAGGGTTACCTGGGTTG | TCACATGCGCCTTGATGTCTG |
|  | *Mus* | GCTCTTACTGACTGGCATGAG | CGCAGCTCTAGGAGCATGTG |
| *CD206* | *Homo* | CTACAAGGGATCGGGTTTATGGA | TTGGCATTGCCTAGTAGCGTA |
|  | *Mus* | CTCTGTTCAGCTATTGGACGC | TGGCACTCCCAAACATAATTTGA |
| *VSIG4* | *Homo* | AGCAGGCAAAGTACCAGGG | TGGCTCCGGTCATCCATCT |
|  | *Mus* | CCTGGGCCACCTAATAGTGC | TGTAGCCTCTCAGGGGATCAT |
| *ACTB* | *Homo* | CATGTACGTTGCTATCCAGGC | CTCCTTAATGTCACGCACGAT |
|  | *Mus* | GGCTGTATTCCCCTCCATCG | CCAGTTGGTAACAATGCCATGT |
| *TGF-β* | *Homo* | TACCTGAACCCGTGTTGCTCTC | GTTGCTGAGGTATCGCCAGGAA |
|  | *Mus* | CTCCCGTGGCTTCTAGTGC | GCCTTAGTTTGGACAGGATCTG |
| *CCL2* | *Homo* | AGAATCACCAGCAGCAAGTGTCC | TCCTGAACCCACTTCTGCTTGG |
| *CCR2* | *Homo* | CCACATCTCGTTCTCGGTTTATC | CAGGGAGCACCGTAATCATAATC |
| Oligo-1 | */* | CAGCCAATGCGATCTAGACTGAATC | GGATCCTACATGTGTACCGTATTCG |
| Oligo-2 | */* | CAGCCAATGCGATCTGGACTGAGTC | GGATCCTACATGCGTACCGCATTCG |
| Homo: *Homo sapiens*; Mus: *Mus musculus*. | | | |
